# Impact of amino acid supplementation on cardiovascular and chronic kidney diseases: a systematic review

**DOI:** 10.1093/cvr/cvag007

**Published:** 2026-01-21

**Authors:** Dustin Mikolajetz, Sahir Kalim, Heidi Noels, Zhuojun Wu, Vera Jankowski, Joachim Jankowski, Sofía de la Puente-Secades

**Affiliations:** Institute of Molecular Cardiovascular Research (IMCAR), Uniklinik RWTH Aachen, Aachen Germany; Department of Medicine, Division of Nephrology, Massachusetts General Hospital and Harvard Medical School, Boston, MA, USA; Institute of Molecular Cardiovascular Research (IMCAR), Uniklinik RWTH Aachen, Aachen Germany; Department of Biochemistry, Cardiovascular Research Institute Maastricht (CARIM), University of Maastricht, Maastricht, The Netherlands; Institute of Molecular Cardiovascular Research (IMCAR), Uniklinik RWTH Aachen, Aachen Germany; Institute of Molecular Cardiovascular Research (IMCAR), Uniklinik RWTH Aachen, Aachen Germany; Institute of Molecular Cardiovascular Research (IMCAR), Uniklinik RWTH Aachen, Aachen Germany; Experimental Vascular Pathology, Cardiovascular Research Institute Maastricht (CARIM), University of Maastricht, Maastricht, The Netherlands; Institute of Molecular Cardiovascular Research (IMCAR), Uniklinik RWTH Aachen, Aachen Germany

**Keywords:** CVD, CKD, Amino acid supplementation

## Abstract

Cardiovascular diseases (CVD) remain the leading cause of mortality worldwide, with chronic kidney disease (CKD) constituting a significant risk factor. Despite the crucial role of amino acids as fundamental dietary components, their impact on the comorbidities of CKD and CVD has been insufficiently studied and warrants greater research attention. Therefore, this systematic review provides a comprehensive overview of the current knowledge regarding the effects of amino acid supplementation on the comorbidities associated with CVD and CKD, as the basis for novel prevention and treatment options. The databases ‘PubMed’ and ‘Web of Science’ were used to perform a literature search on the effects of amino acid supplementation on the comorbidities associated with CVD and CKD. Data synthesis was conducted based on 60 publications, comprising 13 clinical, 43 *in vivo* and four *in vitro* studies. The risk of bias was assessed using three appropriate tools. Studies were classified based on preventive or harmful effects. Altogether, 43 publications reported preventive, and 18 described adverse effects, of which three described both preventive and adverse effects of different amino acids. Only two publications showed no effects caused by amino acids. Arginine and methionine were attributed to the highest number of preventive and adverse effects, respectively. However, a limitation of most publications is the pending translation to humans. Overall, these findings suggest that amino acid supplementation as a potentially valuable addition to treatment options for CVD and CKD patients, although further clinical studies are needed for validation of these findings. This systematic review was funded by the German Research Foundation (DFG, SFB/TRR219) and was registered in the PROSPERO database (CRD42023493924).

## Graphical Abstract

**Graphical Abstract:**
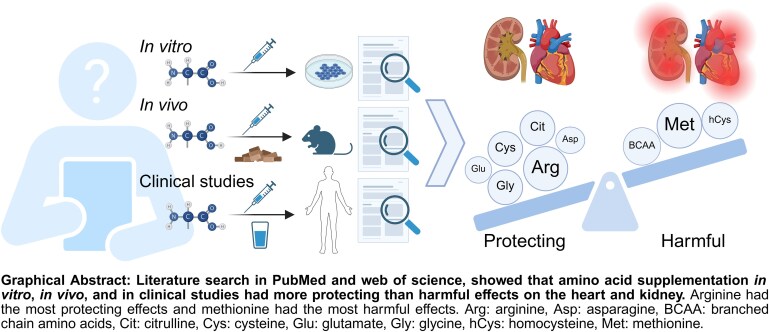

## 1. Introduction

Cardiovascular disease (CVD) remains the major cause of death worldwide^1^ and is projected to remain so despite an expected decline over the next 20 years.^2^ Chronic kidney disease (CKD) remains a major risk factor for CVD,^3,4^ and renal mortality is estimated to increase to be the fifth greatest cause of death by 2040.^2^ Both diseases exacerbate each other through the cardiac-renal and renal-cardiac cross-talk, resulting in increased prevalence of CVD in CKD patients and vice versa.^5,6^ Recently, this cross-talk has received considerable attention, and multiple bidirectional mediators, such as inflammation, oxidative stress, or endothelial dysfunction, have been identified.^7–9^ These mediators promote hypertension, atherosclerosis, renal aging and metabolic disease, which are established comorbidities of CVD and CKD.^10–12^

During the past decades, health-related lifestyle behaviours across developed countries, such as physical activity, sleep duration, energy balance, and dietary habits, have changed incredibly.^13,14^ Generally, the population spends less time on meals and increasingly relies on processed foods, negatively influencing overall health in recent years.^14^ Studies have shown that global red meat consumption increased by 88% from 1990–2018,^15^ even though intake of large amounts of processed or unprocessed red meat is associated with a higher risk for CVD^16^ or CKD.^17^ Amino acids, a key dietary component, especially in red meat, have been insufficiently studied despite their significant impact on physiological and pathological processes. Amino acids can be classified based on their side chain into different subtypes: (i) hydrophilic uncharged amino acids, (ii) hydrophilic cationic amino acids, (iii) hydrophilic anionic amino acids, (iv) sulfur-containing amino acids (SAAs), or (v) hydrophobic amino acids (*Table 1*).^1,8–23^

**Table 1:** Amino acid classification

Amino acid classification	Hydrophilic uncharged amino acids	Hydrophilic cationic amino acids	Hydrophilic anionic amino acids	Sulfur-containing amino acids	Hydrophobic amino acids
Amino acids	Asparagine Glutamine Serine Threonine Citrulline	Arginine Lysine Histidine Homoarginine	Aspartate Glutamate	Cysteine Methionine Homocysteine Taurine	Alanine Glycine Isoleucine Leucine Phenylalanine Proline Tryptophan Tyrosine Valine
Side chain	Polar side chain and uncharged at physiological pH	Polar side chain and positive charged at physiological pH	Polar side chain and negative charged at physiological pH	Contain sulfur atoms in their side chain	Hydrophobic and nonpolar side chain

Amino acids are classified into hydrophilic uncharged amino acids, hydrophilic cationic amino acids, hydrophilic anionic amino acids, sulfur-containing amino acids, and hydrophobic amino acids based on their side chain.

Recent studies have shown that amino acids can protect proteins from harmful post-translational modifications (PTMs) *in vitro*, which are induced by increased uraemic toxins in patients with CKD,^24^ a major driver of CVD in CKD patients.^25–27^ By preventing these PTMs, amino acids provide a compelling link between dietary intake and CKD-related health outcomes.

Since the intake and biosynthesis of amino acids are diet-dependent,^28,29^ the impact of considering amino acid balance in the diet of patients with CVD and CKD is essential.^30^ Especially with regard to the latest guidelines for managing CVD^31^ and CKD,^30^ which focus only on high vs. low protein diets, the role of individual amino acids is overlooked. Therefore, this systematic review aims to give a structured and detailed overview of amino acid supplementation in CVD and CKD, highlighting their preventive and adverse effects, to shift the focus from protein diet to individual amino acids for future guidelines to manage CVD and CKD.

## 2. Methods

### 2.1 Data sources and searches

**Figure 1:**
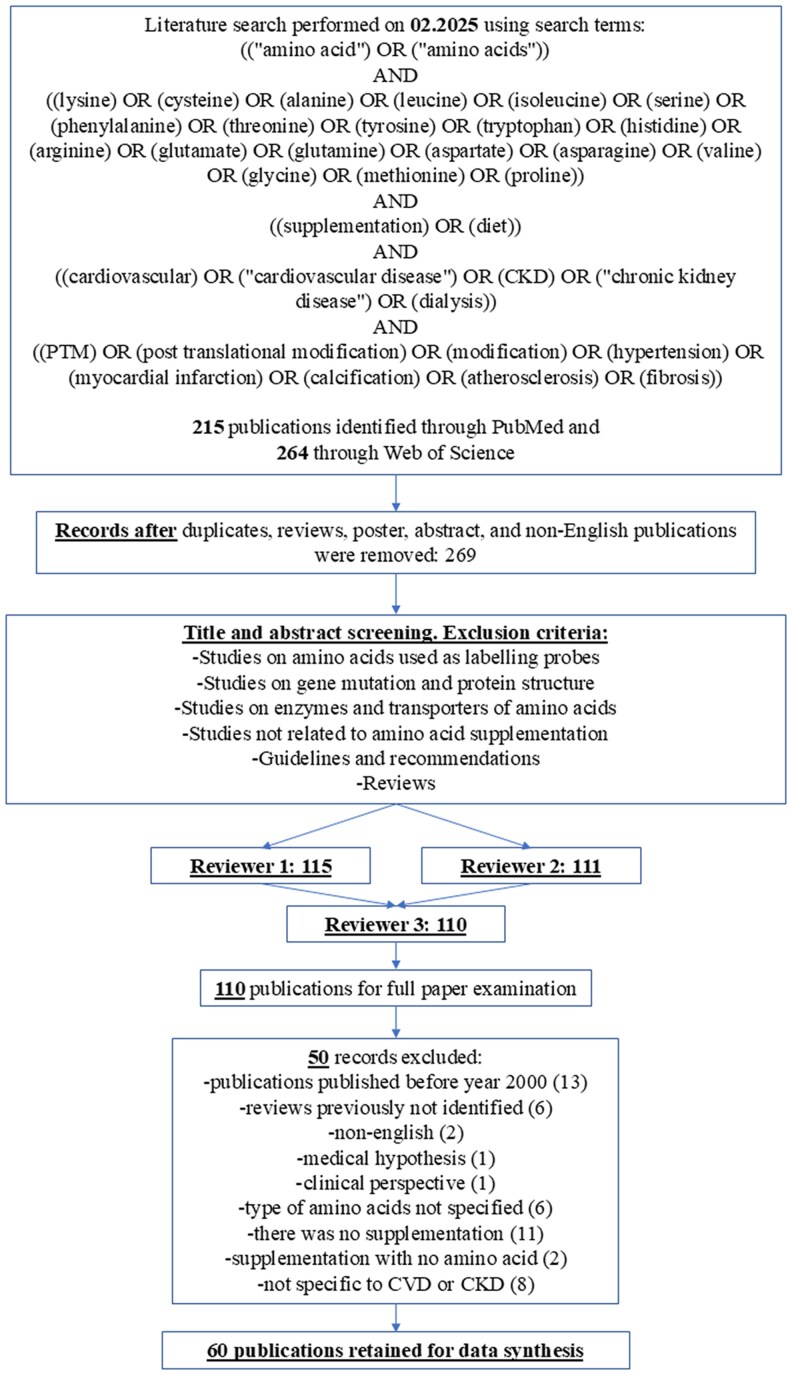
Flow diagram of the systematic search for the effect of amino acid supplementation on cardiovascular and CKD.

The authors performed a literature search in both ‘PubMed’^32^ and ‘Web of Science’,^33^ two of the most important literature databases for biomedical research, servers to identify eligible studies on the 11 February 2025, using the search strategy given in *Figure 1*. The study was registered in the PROSPERO database (CRD42023493924).^34^

In detail, the terms and conditions used for the systematic literature search were as follows: ((‘amino acid’) OR (‘amino acids’)) AND ((lysine) OR (cysteine) OR (alanine) OR (leucine) OR (isoleucine) OR (serine) OR (phenylalanine) OR (threonine) OR (tyrosine) OR (tryptophan) OR (histidine) OR (arginine) OR (glutamate) OR (glutamine) OR (aspartate) OR (asparagine) OR (valine) OR (glycine) OR (methionine) OR (proline)) AND ((supplementation) OR (diet)) AND ((cardiovascular) OR (‘cardiovascular disease’) OR (CKD) OR (‘chronic kidney disease’) OR (dialysis)) AND ((PTM) OR (post-translational modification) OR (modification) OR (hypertension) OR (myocardial infarction) OR (calcification) OR (atherosclerosis) OR (fibrosis)). The ‘Preferred Reporting Items for Systematic Reviews and Meta-Analyses’ (PRISMA) guidelines were followed while composing and drafting the systematic review.

### 2.2 Study selection criteria

Two reviewers (D.M. and S.P.S.) independently identified and evaluated papers by the title for inclusion or exclusion using the conditions described above. A third reviewer (J.J.) was consulted to achieve consensus in case of disagreements.

The two reviewers conducted three iterative rounds of evaluation of the publications, with each successive round involving a progressively more detailed assessment before synthesizing the data. First, duplicates, reviews, posters, abstracts, and non-English publications were removed. Secondly, the title and abstract were screened and excluded based on the following criteria: (i) studies on amino acids used as labelling probes, (ii) studies on gene mutation and protein structure, (iii) studies on enzymes and transporters of amino acids, (v) studies not related to amino acid supplementation, (v) guidelines and recommendations, and (vi) reviews. The final round of screening involved evaluating the full paper based on the following exclusion criteria: publications published before the year 2000, reviews previously not identified, non-English publications, medical hypotheses, clinical perspectives, type of amino acids not specified, publications including amino acids without supplementation, supplementation with no amino acids, and publications not specific to CVD or CKD.

The exclusion of papers prior to 2000 was undertaken due to their low accessibility, and to focus on research within the latest years. Amino acids not specified relate to publications that supplement a mixture of unrelated amino acids, without investigating which amino acid causes the specific effect. Data collection was performed independently by D.M. with supervision of S.P.S., and confirmation by JJ in case of indecisiveness.

### 2.3 Risk of bias assessment

The risk of bias was assessed with three different tools, according to the type of study evaluated: for *in vitro* studies, ‘Quality Assessment Tool For In Vitro Studies' (QUIN Tool),^35^  *in vivo ‘SYstematic Review Centre for Laboratory animal Experimentation' (*SYRCLE),^36^ and for clinical studies, the ‘Modified Downs and Black checklist’^37^ were used (see Supplementary material online, *Table S1*). The assessment was done independently by the reviewers. Before beginning the assessment, the reviewers discussed questions about the tools and evaluated one publication before proceeding with the total evaluation. No studies were removed due to the risk of bias.

### 2.4 Data extraction

To perform data extraction, all publications were ordered by the amino acid(s) analysed, whether a preventive or adverse effect was described and which comorbidity they focused on. Afterwards, the time frame, conclusion, and involved mediators for the mode of action of the studies were extracted. In addition, for the clinical studies, the study duration, study cohort, and main outcome were extracted.

For the clinical studies included in this systematic review, a detailed overview with their type of study, analysed amino acids, study name, durations, populations, amino acid dosage, total intake, and main outcomes are given in Supplementary material online, *Table S6*. The CKD effects encompassed comorbidities that arise after renal function declines, such as an increase in uraemic toxins, PTMs, calcification, and renal aging. The term ‘metabolic disorders’ encompasses obesity and its obesity-related-vascular-dysfunction directly linked to CVD or CKD. Any decrease in the development of the mentioned comorbidities was set up as a preventive effect, while an adverse effect was given after an increase in uraemic toxins or the development of renal aging (by measuring kidney function and kidney parameters).

The term ‘*cardiovascular events*’ was used as a comprehensive term encompassing studies that analysed one of several cardiovascular complications, such as coronary heart disease, stroke, and myocardial injury. The term also included studies on general outcomes, including cardiac remodelling, cardiovascular risk factors, and blood vessel morphology. Overall, this systematic review presents the main outcomes of all 60 included publications, providing as much detail as possible from each publication to illustrate the mode of action analysed in each study. When reported in publications, exact values are provided. Non-significant data are given with their *P*-value when relevant findings were observed. All *in vivo* studies gave a dose compared to the usual human intake, when calculating the human equivalent dose was possible (see Supplementary material online, *Table S5*), only exceptions are stated in the results section.

## 3. Results

### 3.1 Identification of amino acid supplementation studies with impact on the development and progression of CVD and CKD

The databases ‘PubMed’ and ‘Web of Science’ were used for the literature search on 11 February 2025. *Figure 1* gives an overview of the search terms and screening procedure. Initially, 215 publications were identified in ‘PubMed’ and 264 in ‘Web of Science’. After the first round of screening, 269 publications were considered eligible for the systematic review. The next screening involved title and abstract screening, as well as consulting a third reviewer, which led to the eligibility of 110 publications. In the final stage of screening, the complete publications were carefully examined. As a result, 60 publications were considered suitable for detailed assessment and inclusion in the systematic review.

**Figure 2:**
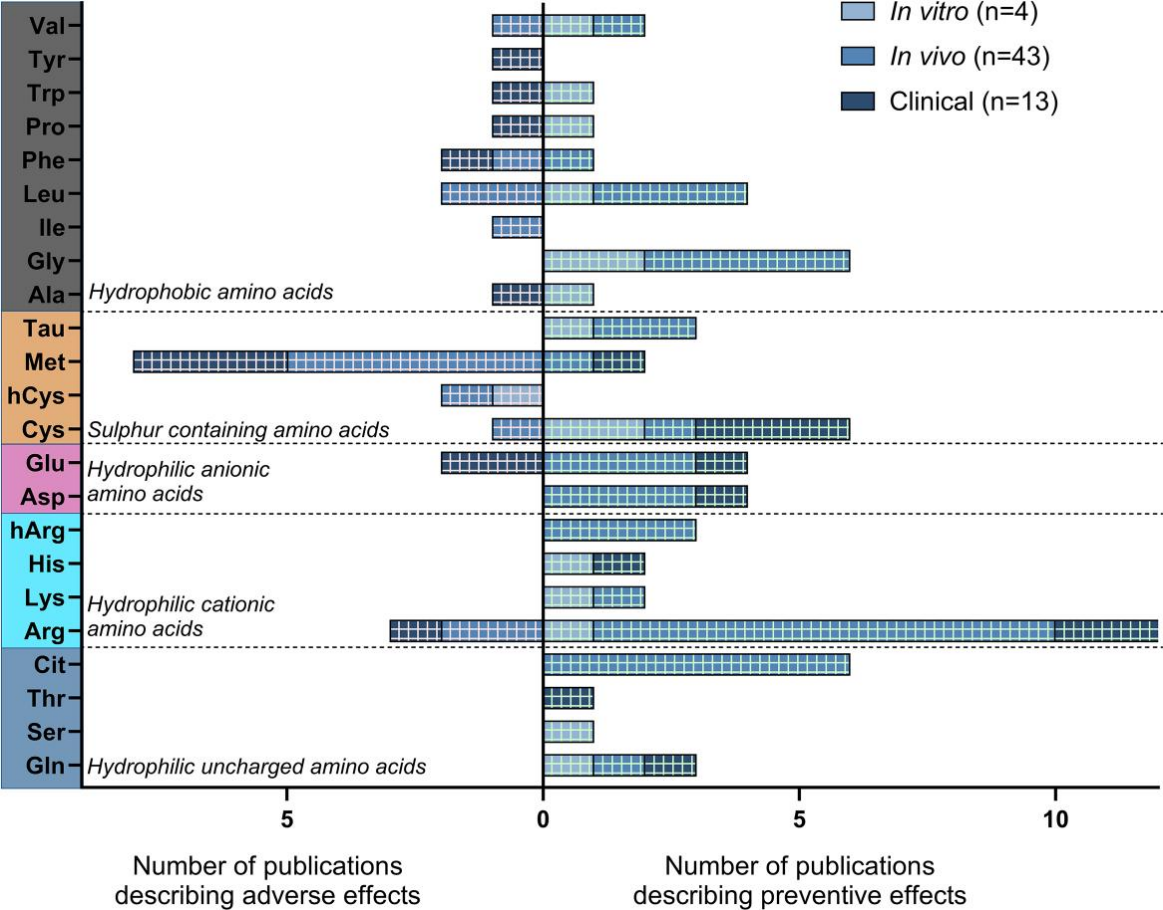
Forest plot of publications describing preventive or adverse effects for each amino acid. Summary of number of publications per amino acid regarding its preventive or adverse effect *in vitro*, *in vivo*, or in clinical studies.

Among these 60 publications, 43 studies focused on *in vivo* experiments, with another 13 studies including clinical data, and only four studies were based on *in vitro* experiments (*Figure 2*). From the 13 clinical studies, only five were ‘randomized controlled trials’ (RCTs). From the seven observational trials, three had a retrospective approach. The last clinical study was an interventional study. In all the clinical studies, amino acid supplementation was not the primary endpoint, but the studies primarily focused on amino acid intake or supplementation. Of these 60 publications, six focused solely on measuring the association between amino acid supplementation and their outcome on comorbidities relevant to CVD or CKD, without further exploration of the underlying signalling pathway.^38–43^ The risk of bias assessment showed a low risk for 17 publications, a medium risk for 43 publications, and no publication with a high risk (see Supplementary material online, *Table S1*).

### 3.2 Identification of amino acids causing preventive and adverse effects on comorbidities of CVD and CKD

**Figure 3:**
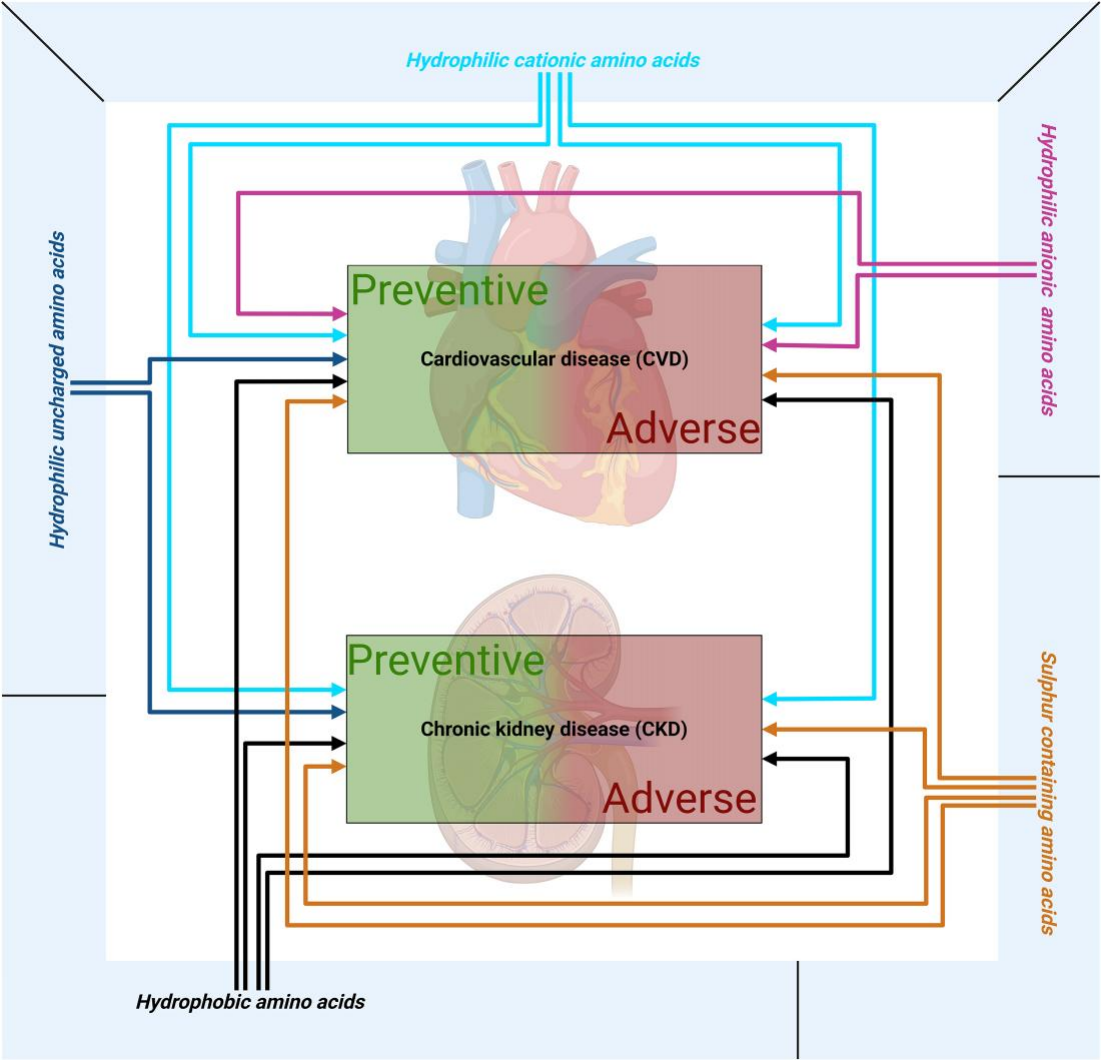
Overview of amino acid groups and their effect on cardiovascular disease and chronic kidney disease. The five amino acid groups: hydrophilic uncharged amino acids, hydrophilic cationic amino acids, hydrophilic anionic amino acids, sulfur-containing amino acids, and *hydrophobic amino acids* and preventive or adverse effects on cardiovascular disease and chronic kidney disease.

The impact of the amino acids was mainly categorized into preventive or adverse effects on CVD and CKD. As shown in *Figure 3*, *hydrophilic uncharged amino acids* have been exclusively associated with preventive effects on CVD and CKD. *Hydrophilic anionic amino acids* showed both preventive and adverse effects only on CVD, whereas *hydrophilic cationic*, sulfur*-containing*, and *hydrophobic amino acids* demonstrated both preventive and adverse effects on CVD and CKD (see Supplementary material online, *Table S2*).

**Figure 4:**
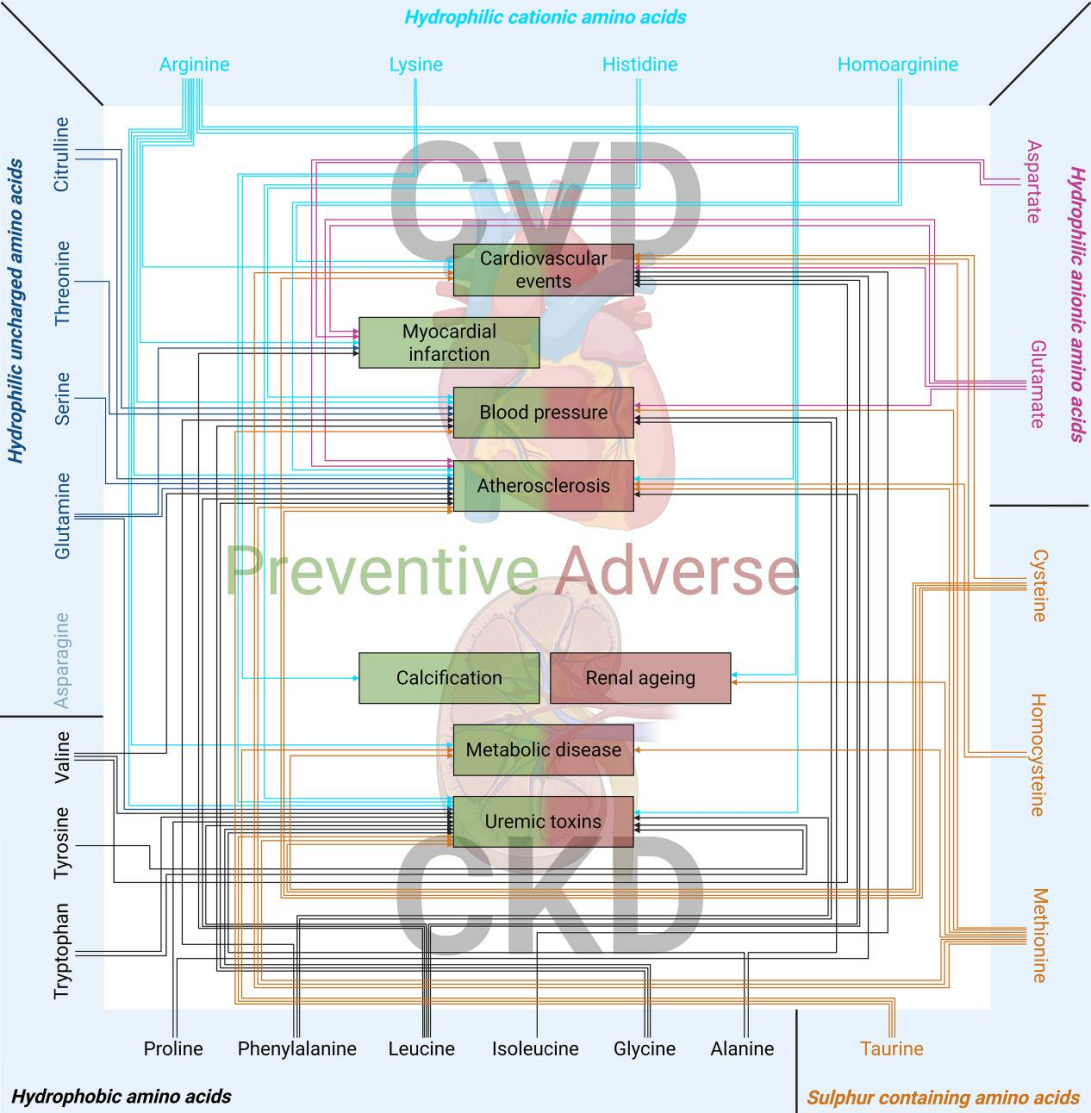
Overview of amino acids and their preventive and adverse effects on comorbidities of cardiovascular disease (cardiovascular events, myocardial infarction, blood pressure, and atherosclerosis) and chronic kidney disease (calcification, renal aging, metabolic disease, and uraemic toxins). Amino acids are divided in groups and connected with arrows to the comorbidities where they have a preventive or adverse effect. No effects were found for the fade-out amino acids.

Based on observed effects in related comorbidities, the findings are summarized in *Figure 4* and Supplementary material online, *Tables S3* and *S4*. After the analysis, 46 preventive and 23 adverse effects were identified (*Figure 4* and Supplementary material online, *Tables S3 and S4*). No studies investigating the effect of asparagine were available at this time.

**Figure 5:**
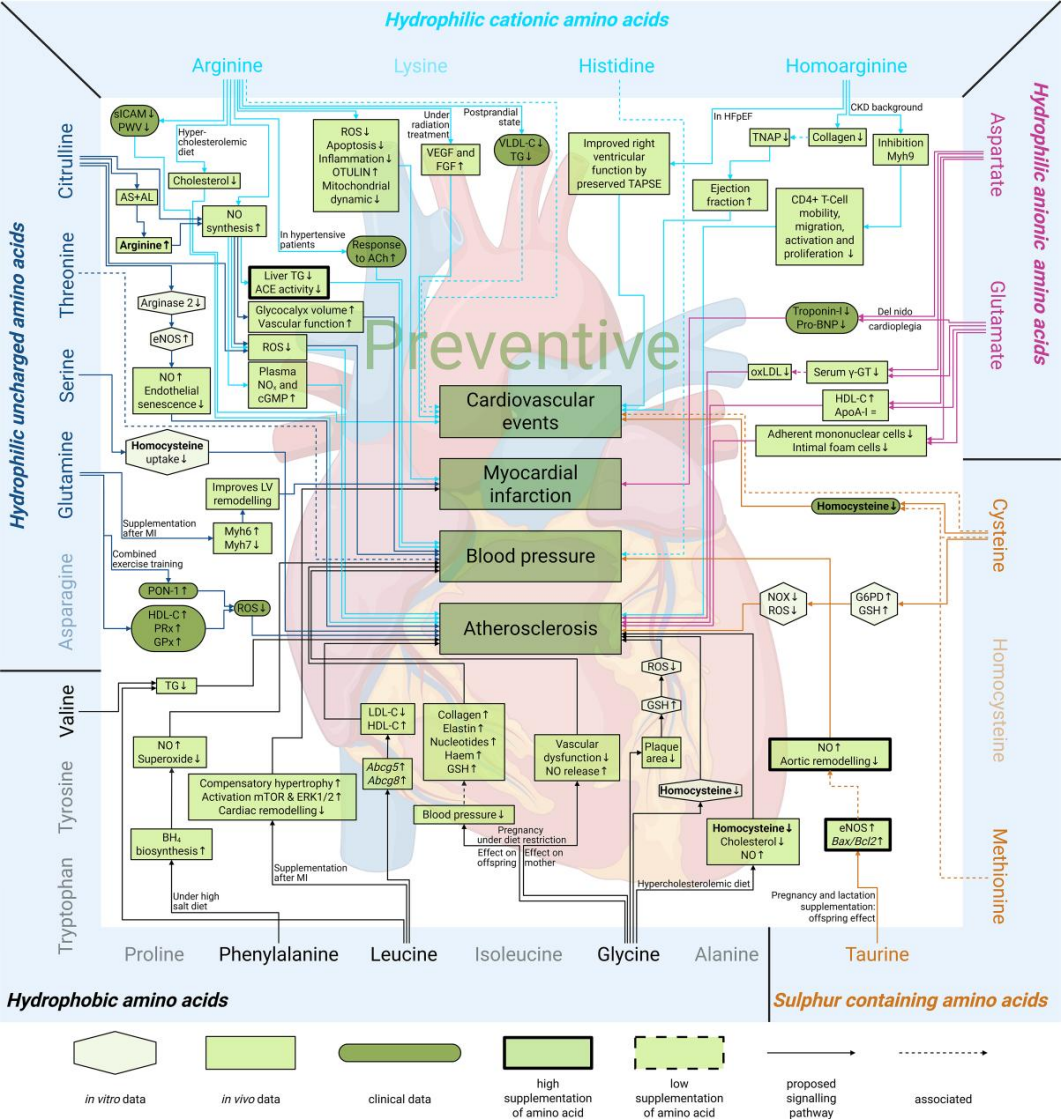
Mechanisms of amino acid supplementation mediating preventive effects on cardiovascular disease: signalling pathways. Amino acids are categorized into distinct groups and linked to the corresponding signalling pathways associated with comorbidities where they exert preventive effects. The shapes and indicate the experimental setting of the study the mechanism is based on: *in vitro* (hexagonal), *in vivo* (rectangle), and clinical (round corners). A thick or dashed border indicates that a human equivalent dose at least 10 times higher (thick) or lower (dashed), respectively, was used. No effects were found for the fade-out amino acids. Continuous lines show the direct effects of amino acids, and dashed lines show associations. *Abcg5:* adenosine triphosphate-binding cassette sub-family G member 5*, Abcg8:* adenosine triphosphate-binding cassette sub-family G member 8, ACE: angiotensin 1 converting enzyme, ACh: acetylcholine, AL: argininosuccinate lyase, ApoA1: apolipoprotein A1, AS: argininosuccinate synthetase, BH_4_: tetrahydrobiopterin, cGMP: cyclic guanosine monophosphate, eNOS: endothelial nitric oxide synthase, ERK: extracellular signal-regulated kinase, FGF: fibroblast growth factor, G6PD: glucose-6-phosphate dehydrogenase, GPx: glutathione peroxidase, GSH: glutathione, HDL-C: high-density lipoprotein-cholesterol, HFpEF: heart failure with preserved ejection fraction, LDL-C: low-density lipoprotein-cholesterol, LV: left ventricular, MI: myocardial infarction, mTOR: mammalian target of rapamycin, Myh: myosin heavy chain, NO: nitric oxide, NO_x_: nitric oxides, NOX: NADPH oxidase, ox-LDL: oxidized low-density lipoproteins, PON-1: paraoxonase, Pro-BNP: pro-brain natriuretic peptide, PRx: total peroxidase, PWV: pulse wave velocity, ROS: reactive oxygen species, sICAM: soluble intercellular adhesion molecule, TAPSE: tricuspid annular plane systolic excursion, TG: triglyceride, TNAP: tissue-non-specific alkaline phosphatase, VEGF: vascular endothelial growth factor, VLDL-C: very low-density lipoprotein-cholesterol, γ-GT: gamma-glutamyl transferase.

**Figure 6:**
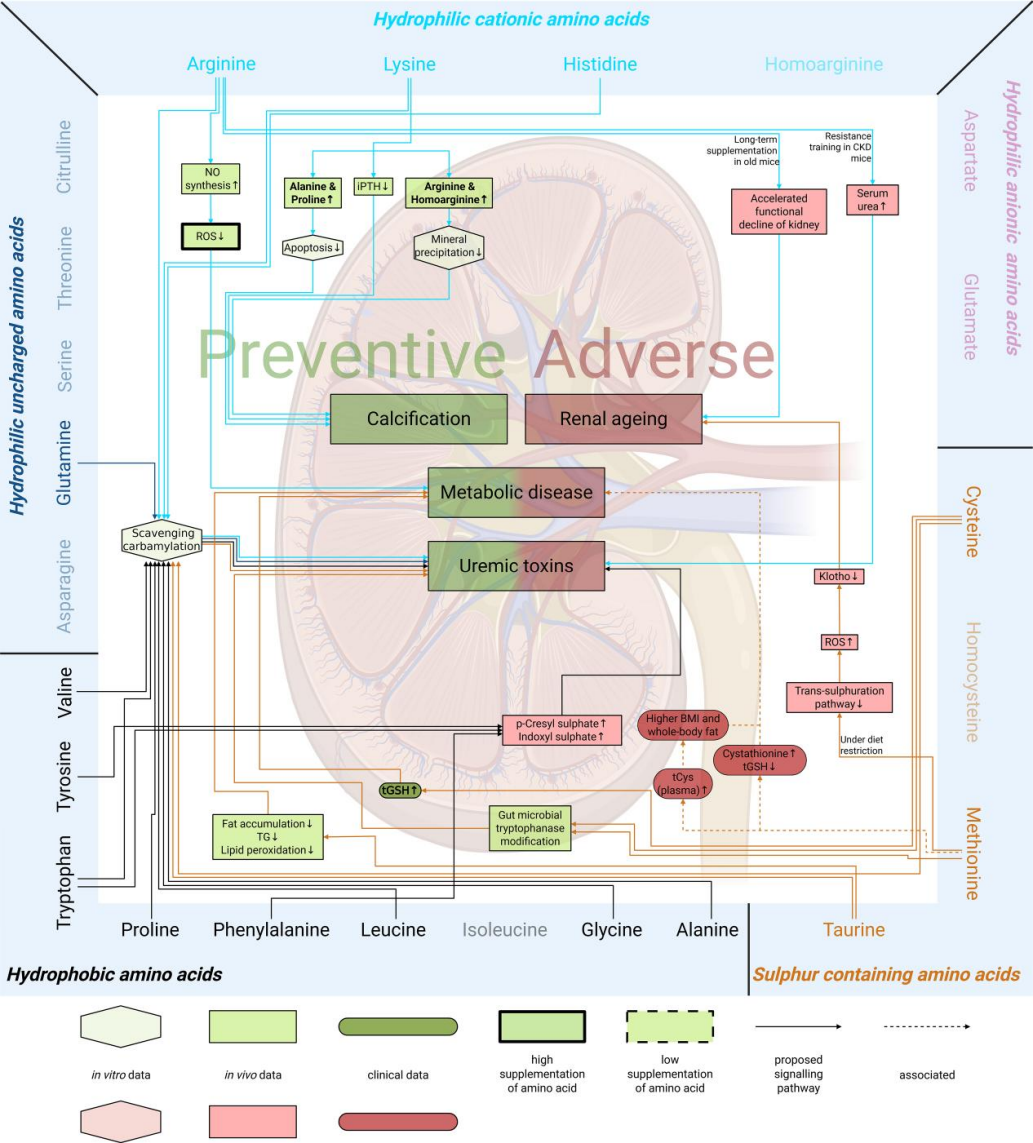
Mechanisms of amino acid supplementation mediating preventive and adverse effects on renal diseases: signalling pathways. Amino acids are categorized into distinct groups and linked to the corresponding signalling pathways associated with comorbidities where they exert preventive or adverse effects. The shapes indicate the experimental setting of the study the mechanism is based on: *in vitro* (hexagonal), *in vivo* (rectangle), and clinical (round corners). A thick or dashed border indicates that a human equivalent dose at least 10 times higher (thick) or lower (dashed), respectively, was used. No effects were found for the fade-out amino acids. Continuous lines show the direct effects of amino acids, and dashed lines show associations. BMI: body mass index, CKD: chronic kidney disease, tCys: total cysteine, tGSH: total glutathione, iPTH: intact parathyroid hormone, NO: nitric oxide, ROS: reactive oxygen species, TG: triglyceride.

**Figure 7:**
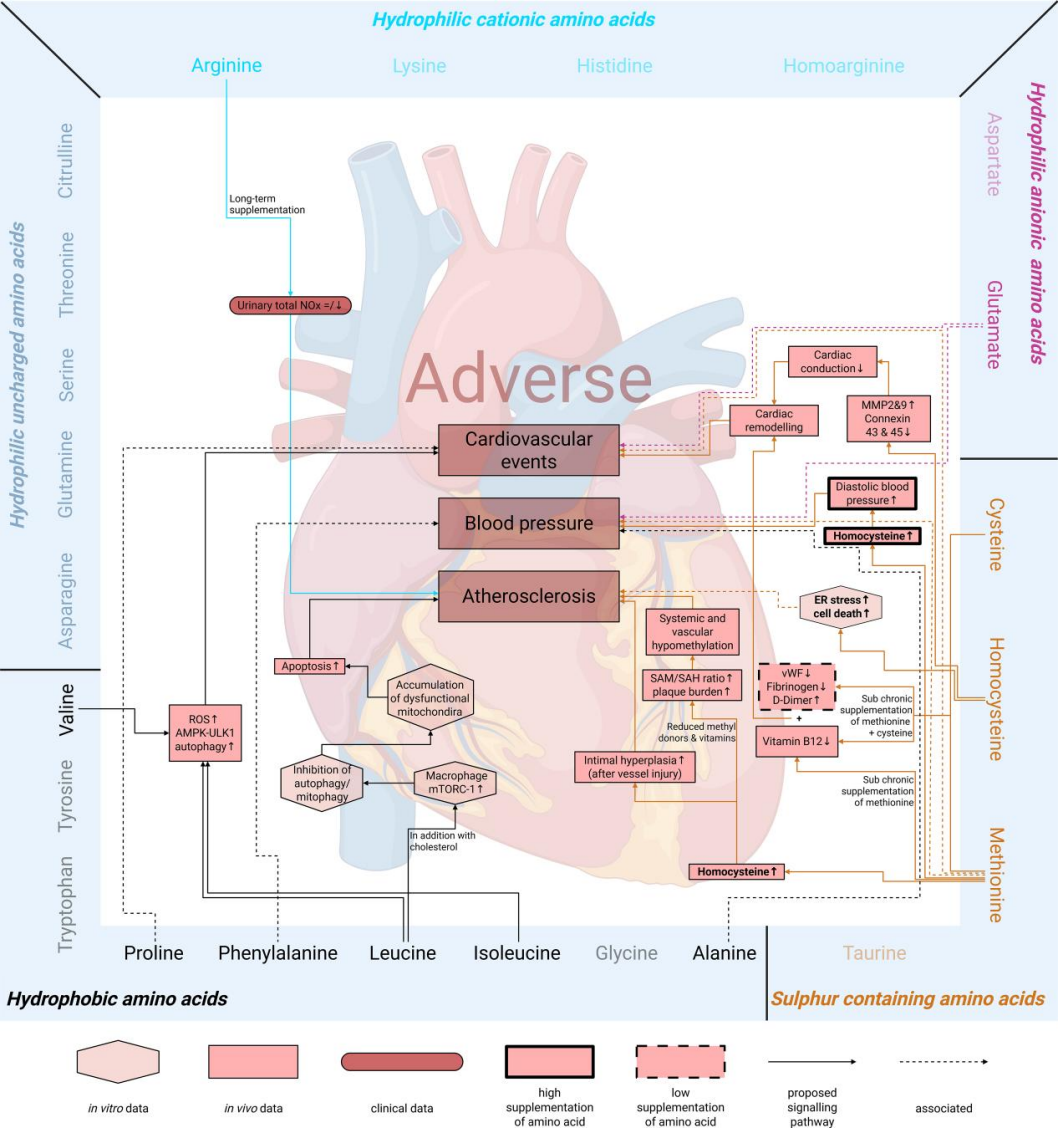
Mechanisms of amino acid supplementation mediating adverse effects on cardiovascular disease: signalling pathways. Amino acids are divided in groups and connected through the corresponding pathway to the comorbidities where they have an adverse effect. The shapes and colour tones indicate the experimental setting of the study the mechanism is based on: *in vitro* (hexagonal), *in vivo* (rectangle), and clinical (round corners). A thick or dashed border indicates that a human equivalent dose at least 10 times higher (thick) or lower (dashed), respectively, was used. No effects were found for the fade-out amino acids. Continuous lines show direct effects of amino acids and dashed lines show association. AMPK: AMP-activated protein kinase, ER: endoplasmic reticulum, MMP: matrix metalloproteinase, mTORC1: mammalian target of rapamycin complex 1, NO_x_: nitric oxides, ROS: reactive oxygen species, SAH: S-adenosylhomocysteine, SAM: S-adenosylmethionine, ULK1: UNC-51 like kinase 1, vWF: von Willebrand factor.

Next, we investigated the signalling pathways described in the eligible publications to explore the preventive (*Figures 5* and *6*; Supplementary material online, *Table S3*) and adverse (*Figures 6* and *7*; Supplementary material online, *Table S4*) effects of amino acid supplementation on CVD and CKD. This step aimed to identify the molecular mechanisms through which amino acid supplementation may influence disease progression, encompassing both preventive and adverse effects. Additionally, we examined how these signalling pathways might offer insights into potential therapeutic targets or biomarkers for the management of CVD and CKD through amino acid supplementation.

### 3.3 Mechanisms of amino acid supplementation causing preventive effects in the context of CVD and CKD

The preventive effects of amino acid supplementation on the development, progression, and prevention of CVD and CKD comorbidities, along with their underlying pathways, are given in *Figure 5, 6* and Supplementary material online, *Table S3*.

### 3.3.1 Hydrophilic uncharged amino acids: preventive effects

The *hydrophilic uncharged amino acid glutamine* has been investigated *in vitro, in vivo*, and in patients. *In vitro*, glutamine prevents carbamylation of albumin by 21%,^24^ which is crucial for maintaining protein function and relieves the stress coming from uraemic toxins. *In vivo*, *glutamine* supplementation in the context of myocardial infarction in mice, leads to an increase in ‘myosin heavy chain 6’ (Myh6) and a decrease in ‘myosin heavy chain 7’ (Myh7) expression in the remote area of the infarcted heart, improving left ventricular (LV) remodelling by increasing the LV anterior and posterior wall thickness and increasing the ejection fraction.^44^ In addition, in a randomized controlled trial *glutamine* supplementation increases the activity of ‘total peroxidase’ (PRx; by 5-fold), ‘glutathione peroxidase’ (GPx; 3.8-fold), and increases ‘high-density lipoprotein-cholesterol’ (HDL-C; +12%) in plasma, as well as, when combined with exercise training, increases the activity of ‘paraoxonase-1’ (PON-1, 1.2-fold).^45^ The increase of these enzyme activities and lipoprotein concentrations suggests a reduced concentration of ‘reactive oxygen species’ (ROS), presumably reducing the oxidation of ‘low-density lipoproteins’ (ox-LDL),^45^ potentially improving the outcome of atherosclerosis. The amino acid *serine* has only been studied *in vitro,* where it decreases the intracellular homocysteine concentration in endothelial cells by competitively inhibiting homocysteine uptake,^46^ a key factor in the development of atherosclerosis.^46^

Dietary *threonine* intake has been studied in patients with prevalent CVD in a RCT and is associated with a reduction in systolic blood pressure (SBP), as indicated by an odds ratio (OR) of 0.84 [95% confidence interval (CI), 0.74–0.96].^41^ Meanwhile, *citrulline* has been studied *in vitro* and *in vivo*^47^: under high glucose conditions, *citrulline* decreases protein expression of ‘arginase 2’ and increases ‘endothelial nitric oxide synthase’ (eNOS) activity *in vitro*, as well as increases ‘nitric oxide’ (NO), and restores endothelial senescence *in vivo*.^47^ Altogether, these effects may reduce the progression of atherosclerosis.

### 3.3.2 Uncharged and hydrophilic amino acids: preventive effects of citrulline and arginine

The *hydrophilic uncharged amino acid citrulline* exhibits similar effects as *the hydrophilic cationic amino acid arginine*. This similarity is attributed to the citrulline/arginine recycling pathway, which converts citrulline by ‘argininosuccinate lyase’ (AL) and ‘argininosuccinate synthetase’ (AS) to arginine.^48^ Arginine, in turn, is converted by the NOS into citrulline.^48^  *Citrulline* has been widely studied in *in vivo* models, where it increases NO synthesis^47–51^ and reduces the production of ROS.^52^ Comparable effects have also been observed *in vitro* and mainly *in vivo* for *arginine*.^47,51,53–56^ Supplementation of *citrulline* during hypoxia decreases pulmonary arterial pressure by an increase in NO levels in piglets forming a separate subcellular pool of arginine exclusively for NO production, which cannot be accurately measured.^48^ Additionally, in preeclampsia-like mice during late pregnancy, *citrulline* increases vascular glycocalyx volume, which is reported to play a critical role in vascular function and NO production, improving NO-dependent relaxation and reducing blood pressure to levels of control animals.^49^ Furthermore, *citrulline* reduces ROS, decreasing blood pressure in a salt-induced hypertension model.^50^ Under intermittent hypoxia, *citrulline* decreases ROS, presumably by reducing the activity of ‘xanthine oxidase’ (XOX), leading to reduced blood pressure and infarct size in rats.^52^

Pre-incubation with *arginine* restores vasodilation in the aortic rings from rats under a low protein diet (38.58 ± 2.71 *E*_min_ vs. 9.03 ± 2.85 *E*_min_), presumably by increasing NO levels due to reverting deficient arginine levels.^56^ In hypertensive rats, arginine supplementation (in a human equivalent dose 10-times higher than the usual daily intake) leads to an increased nitrate concentration in kidney tissue (0.31 ± 0.02 µM/g vs. 0.40–0.47 µM/g), and reduces liver ‘triglyceride’ (TG) levels (27.9 mg/g vs. 20.9–21.6 mg/g) and ‘angiotensin-I converting enzyme’ (ACE) activity (92.04 ± 5.5 nmol/mg/min vs. 10.3–37.56 nmol/mg/min), thus reducing SBP (146.7 ± 4.5 mmHg vs. 126.6–127.5 mmHg).^53^ Arginine supplementation increases NO levels and reduces ROS,^54,55^ which was accompanied with reduced obesity-related changes in the heart, liver, and pancreas of rats,^54^ using a human equivalent dose 10-times higher than the usual daily intake.^54^ Furthermore, the combination of *arginine* with antioxidants has been shown to reduce atherosclerotic plaque area in mice (254.4 ± 34.5 vs. 195.3 ± 28.8 µm^2^ ×10^3^), suggesting a possible synergistic effect that enhances vascular health and mitigates oxidative stress, which is a key contributor to atherosclerosis.^55^

Supplementing *citrulline* and *arginine* under high glucose conditions enhances eNOS activity *in vitro* and increases NO production in rats. This boost in NO restores endothelial function and delays cellular senescence in rats,^47^ reducing factors involved in the pathogenesis of atherosclerosis.^47^ The combined supplementation also leads to a more rapid increase in plasma arginine concentration than the single amino acids supplementation, and thus increases plasma nitric oxides (NO_x_) as well as cyclic guanosine monophosphate (cGMP), improving the vascular flow in rabbits.^51^

#### 3.3.3 Hydrophilic cationic amino acids: preventive effects

*Arginine*, *lysine*, *histidine*, and *homoarginine* belong to the group of *hydrophilic cationic amino acids.* Apart from what has been mentioned before, *arginine,* when combined with a hypercholesterolaemic diet, reduces cholesterol levels in plasma (138.4 ± 19.8 mg/dL vs. 93.7 ± 6.5 mg/dL) and liver (31.5 ± 3.1 mg/g vs. 25.7 ± 2.9 mg/g) in rats, presumably by activating lipases and improving lipid metabolism in the liver and plasma,^57^ improving parameters affecting the risk for cardiovascular events.^57^ In addition, in a myocardial infarction model, *arginine* reduces the production of ROS, apoptosis [reducing apoptosis regulator (Bcl-2 associated X protein (BAX)) and caspase-3], inflammation (reducing TNFα and IL-1β plasma concentration), and mitochondrial dynamics (Mfn-2 and Drp-1 reduction) in the heart tissue of mice. Furthermore, *arginine* supplementation increases otulin levels in the heart tissue, suppressing cell death by cleaving methionine-1 (M1)-linked polyubiquitin chains. This mechanism promotes cardioprotective effects and might mitigate pathophysiological processes in myocardial infarction.^58^ Moreover, after radiation treatment in rats, *arginine* prevents the significant decrease of ‘vascular endothelial growth factor’ (VEGF) and ‘fibroblast growth factor’ (FGF), thereby preventing a reduction of arterial wall thickness, overall preserving the morphology and density of blood vesseles.^59^  *Arginine* supplementation has been studied in a randomized control trial in healthy young humans, where it reduces inflammatory processes by decreasing ‘soluble intercellular adhesion molecule’ (sICAM) and increasing vascular elasticity, evaluated by the reduction of the ‘pulse wave velocity’ (PWV), after acute smoking.^60^

In a clinical study with hypertensive patients and subjects with a positive history of hypertension with reduced arginine transport, intra-arterial *arginine* administration increases forearm blood flow after acetylcholine (ACh) infusion, possibly by increasing the bioavailability of NO.^61^ Furthermore, in another RCT, *arginine* decreases ‘very low-density lipoprotein-cholesterol’ (VLDL-C) and TG in a post-prandial state, attenuating possibly cardiovascular risk factors.^62^

*Lysine’*s preventive effect has been reported *in vivo*. It reduces vascular calcification in the CKD rat model by decreasing intact parathyroid hormone (iPTH) levels, due to increased serum calcium levels.^63^ In addition, lysine also reduces vascular calcification by increasing alanine (from 346.5 ± 100.1 nmol/mL to 473.7 ± 126.7 nmol/mL), proline (from 120.6 ± 29.8 nmol/mL to 173.4 ± 43.1 nmol/mL), arginine (from 91.3 ± 18.9 nmol/mL to 125.6 ± 28.0 nmol/mL), and homoarginine concentrations (from 4.4 ± 0.7 nmol/mL to 6.5 ± 3.0 nmol/mL) in plasma. *Alanine and proline* reduce Ca/P-induced apoptosis *in vitro*, while arginine and homoarginine reduce mineral precipitation.^63^

The amino acid *histidine* is associated with decreased blood pressure in patients with prevalent CVD.^41^  *Homoarginine* supplementation in rats for 26 weeks improves right ventricular function by preserving ‘tricuspid annular plane systolic excursion’ (TAPSE; from 1.2 ± 0.3 mm to 1.7 ± 0.3 mm) in ‘heart failure with preserved ejection fraction’ (HFpEF),^64^ and has been shown to reduce cardiac remodelling by reducing collagen deposition (from 5.3 ± 0.5% to 3.6 ± 0.2%), possibly acting as a specific non-competitive inhibitor of the ‘tissue-non-specific phosphatase’ (TNAP), leading to an improved ejecting fraction (from 52 ± 2% to 64 ± 2%) and reduced myocyte cross-sectional area in 5/6 nephrectomy rats (from 263 ± 4 µm^2–^233 ± 4 µm^2^).^65^ Both mechanisms might contribute to reduce the risk of cardiovascular events.^64,65^ Furthermore, *homoarginine* supplementation in mice leads to inhibition of ‘myosin heavy chain 9’ (Myh9) in T-cells, reducing mobility, migration, activation, and proliferation, thereby reducing atherosclerotic lesion volume (−24%).^66^ The *hydrophilic cationic amino acids arginine*, *lysine*, and *histidine* protect albumin from carbamylation *in vitro* by 34%, 23%, and 35%, respectively,^24^ reducing the systemic effect of uraemic toxins.

### 3.3.4 Hydrophilic anionic amino acids: preventive effects

The *hydrophilic anionic amino acids aspartate* and *glutamate* were always supplemented together. *In vivo,* when given with cholesterol, they induce anti-atherogenic effects in rabbits^67–69^ by reducing the formation of fatty streaks and foam cell formation,^67^ presumably due to increasing HDL-C (from 14.7 ± 1.5 mg/dL to 19.6 ± 1.7 mg/dL) while keeping the level of ‘apolipoprotein A-I’ (ApoA-I; from 25.1 ± 0.7 mg/dL to 21.1 ± 1.8 mg/dL) constant.^68^ In addition, *aspartate* and *glutamate* reduce serum ‘gamma-glutamyl transferase’ (γ-GT; from 51.5 ± 9.8 U/L to 31.9 ± 6.3 U/L), thereby supposedly preventing the generation of ox-LDL.^69^ The supplementation of both amino acids together in an enriched ‘del Nido cardioplegia’ solution, in a randomized control trial with patients undergoing a coronary artery bypass surgery, reduces the accumulation of leukocytes with reduced release of troponin-I and ‘pro-brain natriuretic peptides’ (Pro-BNP) levels in blood. This increases LV function, improving the stroke work index (21.5 ± 4.2 units to 33.9 ± 2.6 units at the second postoperative hour) and cardiac index (from 2.4 ± 0.3 L/min/m^2–^3.1 ± 0.8 L/min/m^2^) and is hypothesized to decrease perioperative myocardial infarction.^70^

### 3.3.5 Sulfur-containing amino acids: preventive effects

The SAAs *cysteine* and *taurine* protect albumin *in vitro* from carbamylation by 42 and 46%, respectively.^24^ Furthermore, *in vitro cysteine* increases ‘glucose-6-phosphate dehydrogenase’ (G6PD) activity and ‘glutathione’ (GSH) levels, which is linked to reduced ‘nicotinamide adenine dinucleotide phosphate (NADPH) oxidase’ (NOX) activity and ROS concentration, and thus reducing key factors of atherosclerosis development.^71^  *Cysteine* and *methionine* post-translational modify gut microbial tryptophanase of mice (S-sulfhydration), resulting in a reduced production of indole, a uraemic toxin.^72^ Furthermore, *cysteine* intake has been studied in an observational clinical study showing a trend to reduce plasma homocysteine and is positively associated with GSH levels, thus potentially preventing cardiovascular events and metabolic diseases, respectively.^40^ Moreover, in another observational study, high *cysteine* intake in women reduces the risk of stroke.^43^ The amino acid *taurine* has been studied *in vivo,* when given with monosodium glutamate in rats, it reduces perigonadal fat deposition, plasma levels of TG and ‘malondialdehyde’ (MDA), a marker for lipid peroxidation^73^ possibly reducing the development of metabolic disease. Additionally, administering *taurine* to spontaneously hypertensive rats during pregnancy and lactation, reduces SBP in adult offspring by increasing sex-specific gene expression of eNOS and ‘Bax/Bcl-2’, presumably increasing NO production and preventing aortic remodelling, leading to reduced blood pressure,^74^ although in this study a human equivalent dose 10-times higher than the usual daily intake of taurine was used. Finally, an observational-retrospective study showed that consumption of SAAs, combined with a high-protein diet, leads to lower homocysteine concentration (from 18.6 ± 2.9 µmol/L to 10.8 ± 0.7 µmol/L) in human plasma, which is associated with a reduced risk for cardiovascular events.^38^

### 3.3.6 Hydrophobic amino acids: preventive effects

Among the *hydrophobic amino acids*, *alanine*, *glycine*, *leucine*, *proline*, *tryptophan*, and *valine* act as scavenging agents against cyanate, thereby preventing carbamylation of albumin *in vitro* by 20, 23, 10, 16, 21, and 18%, respectively.^24^ The *hydrophobic amino acid glycine* reduces *in vitro* homocysteine concentration.^46^ In glycine-depleted media, glycine supplementation increases GSH and reduces ROS levels,^75^ suggesting this as the link to its observed ability to reduce the plaque area in an atherosclerotic mouse model.^75^ In combination with a hypercholesterolaemic diet, *glycine* supplementation in rats reduces liver and plasma cholesterol, as well as plasma homocysteine, and increases plasma nitrite/nitrate levels (a marker for NO levels),^57^ improving risk factors for the development of atherosclerosis and cardiovascular events.^57^ When supplementing *glycine* in rats during pregnancy under diet restriction, it reverses impaired relaxation to ACh and isoprenaline, and increases NO release of small mesenteric arteries at the basal level and after ACh-induced relaxation.^76^ Moreover, *glycine*, when supplemented with a low protein diet in pregnant rats, reduces blood pressure in the offspring, potentially due to its role in synthesizing collagen, elastin, nucleotides, haem, and GSH.^77^

Regarding *leucine*, *isoleucine,* and *valine*, which are also called branched-chain amino acids (BCAA), only *in vivo* studies have been reported. *Leucine* increases the expression of *Abcg5* (1.3-fold) and *Abcg8* (0.9-fold) in mice, causing cholesterol efflux from the liver, resulting in a decrease in serum LDL-C (−41.2%) and an increase in HDL-C (+40.2%), thereby decreasing inflammation (monocyte chemoattractant protein-1 levels) and improving the lipid profile, giving a potential link to the observed reduction of aortic atherosclerotic lesion area (−57.6%).^78^ Moreover, when given to post-myocardial infarction in mice, *leucine* activates the ‘mammalian target of rapamycin’ (mTOR), leading to activation of ‘extracellular signal-regulated kinase ½’ (ERK1/2) after 3½ days post-myocardial infarction and causes induction of compensatory hypertrophy and reduces cardiac fibrosis and inflammation, leading to reduced cardiac remodelling and mortality.^79^ When given with a high-cholesterol diet in rats, *leucine* and *valine* decrease blood TG levels (from 60.6 ± 4.9 mg/dL to 47.6 ± 2.1 mg/dL and 39.7 ± 1.3 mg/dL, respectively), reducing risk factors for the pathogenesis of atherosclerosis.^80^ In rats on a high-salt diet, *phenylalanine* supplementation increases BH_4_ in the vasculature and kidneys, thereby causing the NOS to produce less superoxide and more NO, ultimately leading to more vascular relaxation induced by ACh and decreasing SBP (−18 mmHg).^81^

Altogether, 43 publications describe preventive effects caused by amino acid supplementation regarding CVD and CKD. Observations were mainly made *in vivo*, with some supporting *in vitro* and clinical studies.

### 3.4 Adverse mechanisms of amino acid supplementation on CVD and CKD

*Figures 6* and *7* (see Supplementary material online, *Table S4*) summarize the adverse effects of amino acid supplementation and the underlying signalling pathways. Until now, no adverse effects have been reported for *hydrophilic uncharged amino acids*.

### 3.4.1 Hydrophilic cationic amino acids: adverse effects

The *hydrophilic cationic amino acid arginine* has been associated with adverse effects when long-term supplemented *in vivo* or in peripheral arterial disease (PAD). Specifically, long-term *arginine* supplementation leads to an increase in the urinary albumin–creatinine ratio, a marker for renal damage, in old female mice independently of arginase-II, promoting renal aging.^82^ The study by Souza *et al.* shows that *arginine* supplementation in a CKD mouse model with resistance training increased serum urea levels, presumably by promoting a proinflammatory environment and fibrosis in the kidney, thereby reducing kidney function.^83^ In an RCT with patients with PAD, a form of atherosclerosis, long-term arginine supplementation either reduces or fails to improve urinary total NO_x_ (placebo: from 396.7 ± 320.7 µmol/L to 472.6 ± 339.0 µmol/L, L-arginine: 384.5 ± 362.3 µmol/L to 376.5 ± 250.2 µmol/L) levels. Additionally, *arginine* was less effective than placebo in enhancing walking distance (absolute change from baseline: placebo 78 ± 16 m vs. L-arginine: 36 ± 17 m, *P* = 0.09).^84^

### 3.4.2 Hydrophilic amino acid: adverse effects

In humans, the *hydrophilic anionic amino acid glutamate* is associated with increased cardiovascular events [hazard ratio (HR) = 1.30, 95% CI = 1.03–1.64],^39^ and increased blood pressure (SBP OR of 1.13 (95% CI, 1.00–1.28).^41^

### 3.4.3 Sulfur-containing amino acids: adverse effects

The supplementation of the sulfur*-containing amino acid cysteine,* combined with a sub-chronic supplementation of *methionine* in rats, decreases vitamin B12, fibrinogen, the procoagulant factor ‘von Willebrand factor’ (vWF), as well as increases D-dimer in plasma. In comparison, sub-chronic supplementation of *methionine* alone leads to a decrease in vitamin B12 (from 882.0 ± 32.0 to 742.5 ± 25.7 ng/L) and a decrease in vWF activity (from 27.3 ± 11.2–4.2 ± 0.2). The amino acids were supplemented via intraperitoneal injection, while using comparable or 10-times lower human equivalent dose than the usual daily intake for methionine or cysteine, respectively. Both groups showed dilation of the microcirculation and extravasation of erythrocytes; moreover, the methionine + cysteine group also showed intercellular edema.^85^

*Homocysteine* increases ‘endoplasmic reticulum’ (ER) stress, thereby activating the unfolded protein response and causing endothelial cell death *in vitro*, suggesting a possible interaction between homocysteine levels and the development of atherosclerosis^46^; however, validation in higher models is necessary. Additionally, elevated homocysteine plasma levels in mice increase the expression of ‘matrix metalloproteinases’ (MMP) 2 and 9, suggesting extracellular matrix remodelling, while decreasing the levels of ‘connexin proteins 43’ and ‘45’ in myocardial tissue. These connexins are essential components of cardiac gap junctions that facilitate proper electrical signalling between heart cells. Their disruption might contribute to the observed prolonged PR and QRS interval in the homocysteine group.^86^

*Methionine*, which is a direct precursor of homocysteine after various methylation processes, in sham-operated rats, causes hyperhomocysteinemia (from 6.8 ± 0.6 µM to 37.1 ± 9.2 µM) and increases SBP within 3 weeks, as well as changes the diastolic blood pressure (DBP), promoting hypertension,^87^ although a human equivalent dose of 20-times higher than the normal intake was used.^87^ Furthermore, when *methionine* is given with reduced methyl donors and vitamins, it decreases the ‘s-adenosylmethionine’ (SAM) and ‘s-adenosylhomocysteine’ (SAH) ratio in plasma, aorta, and liver as well as increases brachiocephalic artery atherosclerotic plaque burden in mice.^88^ In addition, *methionine* supplementation increases intimal hyperplasia in rats (intima/media increased from 0.2 ± 0.1–1.1 ± 0.2) after vessel injury, presumably due to increased homocysteine values (from 5.4 ± 0.3 µM to 32.8 ± 3.0 µM),^89^ thereby suggesting a link between methionine intake and atherosclerosis. Moreover, under diet restriction in aged mice, *methionine* supplementation inhibits the trans-sulfuration pathways, presumably via downregulation of ‘cystathionine β-synthase’ (CBS), thereby promoting oxidative stress and resulting in lower Klotho expression and consequently accelerating renal aging.^90^ Furthermore, *methionine* supplementation is associated with a trend in increasing plasma total cysteine and a decrease in total plasma glutathione; the former is associated with higher body mass index (BMI) and whole-body fat, all being associated with metabolic disorders in an observational study.^40^ In other observational studies, *methionine* has also be found to be associated with hypertension in patients with prevalent CVD [SBP OR 1.29 (95% CI, 1.14–1.46); DBP OR 1.21 (95% CI, 1.05–1.39)]^41^ and cardiovascular events [hazard rate ratio of the three highest quarters of energy-adjusted methionine intake: 1.31 (95% CI, 0.92–1.86), 1.31 (95% CI, 0.88–1.96), and 2.08 (95% CI, 1.31–3.29) as compared to the lowest quarter].^42^

### 3.4.4 Hydrophobic amino acids: adverse effects

*In vitro leucine* was shown to activate the ‘mammalian target of rapamycin complex-1’ (mTORC-1) in macrophages,^91^ hypothesized to be the main driver in mice fed with high-protein and cholesterol diet to inhibit autophagy/mitophagy and thereby preventing the removal of dysfunctional mitochondria.^91^ The accumulation of dysfunctional mitochondria triggers macrophage apoptosis, promoting plaque complexity and atherosclerosis.^91^ The combination of *hydrophobic amino acids isoleucine*, *leucine,* and *valine in vivo* increases ROS, as well as ‘adenosine monophosphate-activated protein kinase’ (AMPK) and ‘UNC-51-like kinase-1’ (ULK-1) pathway, leading to excessive autophagy in myocardial tissue of mice, promoting myocardial injury and thereby promoting cardiovascular events.^92^ In an observational clinical study, *alanine* and *phenylalanine* are associated with high blood pressure in patients with prevalent CVD [alanine: SBP OR 1.17 (95% CI, 1.05–1.30); DBP OR 1.22 (95% CI, 1.07–1.38); and phenylalanine: DBP OR 1.14 (95% CI, 1.02–1.28)],^41^ in another observational study *proline* is associated with increased risk for cardiovascular events (HR = 1.33, 95% CI = 1.10–1.60).^39^

In mice, *phenylalanine*, *tryptophan,* and *tyrosine* increase the concentration of the uraemic toxins p-cresyl sulfate and indoxyl sulfate, and promote renal function decline as well as kidney fibrosis.^93^ Moreover, in CKD patients stages 4–5, an observational study has shown that while there was no correlation between phenylalanine, tyrosine, and tryptophan to indoxyl sulfate or p-cresyl sulfate, the protein-fibre index was positively correlated to an increased total p-cresyl sulfate (*r* = 0.43, *P* = 0.005) and indoxyl sulfate (*r* = 0.40, *P* = 0.012) concentration.^94^

In total, we found 18 papers describing adverse effects of amino acid supplementation regarding CVD and CKD. Most studies investigated the effects of amino acid supplementation through *in vivo* experiments, and their results have been confirmed by several clinical studies.

### 3.5 Neutral effects of amino acids

Two *in vivo* studies showed no significant effect on the outcome of amino acid supplementation. Schwartz *et al.* showed that when given a hypercholesterolaemia diet to rats, *arginine* supplementation does not affect serum cholesterol levels.^95^ Biswas *et al.* observed that *glycine* supplementation in male mice leads to a reduction of HDL-C (from 34 ± 1 mg/dL to 29 ± 2 mg/dL) and TG (from 58 ± 4 mg/dL to 44 ± 3 mg/dL), but did not lead to a significant reduction in aortic lesions.^96^

### 3.6 Amino acids not studied

*Asparagine* is the only amino acid which has not been studied in any publications reviewed. Therefore, no statements can be made about the possible preventive or adverse effects of asparagine, and first studies are required to make any possible predictions.

## 4. Discussion

CVD and CKD are major global diseases, with CVD leading the cause of death worldwide.^1,97^ In the upcoming 20 years, it is predicted that CVD will still be the leading cause of death worldwide, and CKD will increase and become the fifth.^2^ Since both diseases are closely linked to each other,^3,4^ finding a treatment to tackle both diseases simultaneously would be a high clinical advantage. With the rising interest in amino acids in recent years, due to their effect of scavenging carbamylation, a modification often seen in CKD patients,^24,98^ and their functionality as precursors of important mediators like NO,^39^ they are in an intriguing position to fulfil this role possibly.

The search terms and evaluation by the reviewers resulted in a total of 60 publications addressing the preventive and adverse effects of amino acid supplementation on the cardiovascular and renal system. Overall, 46 preventive and 23 adverse effects (*Figure 4*) were described and attributed to specific amino acids. Most of the studies found are related to *in vivo* experiments, with only a few *in vitro* and some clinical studies (from those only five RCTs). Moreover, some of the current findings need special attentions since some data are based on a combination of amino acids. Furthermore, some (groups of) amino acids cause at the same time preventive and harmful effect.

The *hydrophilic uncharged amino acids* were noticeable as this was the only group causing exclusively preventive effects. Within them, *citrulline* had the highest number of preventive effects although only *in vivo* studies have been reported. However, among all five groups, *arginine* was the amino acid attributed with the majority of preventive effects, including both *in vivo* and clinical studies (*Figure 5–6*; Supplementary material online, *Table S3*). *Arginine* and *citrulline* cause a large number of synergistic effects, as they are tightly involved in the arginine/citrulline recycling cycle, and can be converted into each other.^48^ Most of their preventive effects are caused by an increase in NO synthesis^47–51,56,58,59^ (being NO a key regulator of cardiovascular health). It is important to notice that the supplementation of citrulline *in vivo,* compared to the supplementation of arginine alone, is highly efficient in enhancing the preventive effects of arginine on atherosclerosis, as it improved the bioavailability of arginine, leading to increased NO biosynthesis.^47,51^ Since the increase in NO production is one of the main mediators of arginine supplementation, the addition of citrulline may also further improve the outcome on cardiovascular events, hypertension, and metabolic disease, which was already observed for arginine alone.^53,54,56,59^ Therefore, future studies planning to analyse the effect of arginine supplementation might benefit from considering a combined supplementation of arginine with citrulline.

The studies supplementing the *hydrophilic anionic amino acids* glutamate and aspartate together highlight their potential benefits by reducing the development of atherosclerosis *in vivo* and myocardial infarction in patients^68–70^ but it would be important to investigate their effects when supplemented separately. Understanding how each amino acid independently influences biological pathways could provide deeper insights into their function and determine whether their combined supplementation is synergistic or if they exert distinct and separate effects. The resulting data might lead to more targeted and effective therapeutic approaches, allowing for more precise modulation of specific pathways in cardiovascular and renal diseases. On the other hand, in patients, only *glutamate* has been associated with an increased risk for cardiovascular events^39^ and increased SBP,^41^ but *aspartate* had no significant effect on the cardiovascular events,^39^ underscoring the need for studies to clarify these dual effects in more detail.

Some amino acids, belonging to different groups, like *cysteine* and *glycine,* cause preventive effects by modulating similar mediators, like e.g. increasing glutathione and decreasing homocysteine levels.^40,46,71,75^ However, these results have to be validated in a clinical setting for glycine in the future.

Most number of adverse effects was caused by SAAs, followed by *hydrophobic amino acids*. One major drawback is the partially unclear mechanisms through which these two groups adversely affect blood pressure and cardiovascular events, even though their effect have been seen in observational studies, many effects have only been described by associations with no direct mechanism so far.^39–42^ Among the SAAs*, methionine* showed the highest number of adverse effects. Methionine is a precursor of SAM, an important methyl donor for DNA, RNA, and proteins.^88^ At the same time, SAM gets converted to SAH and then to homocysteine,^40,42,88,89^ which can be metabolized, requiring vitamins as cofactors. This fact should be considered since the adverse effect of methionine might be exacerbated when combined with a vitamin-deficient diet.^88^ On the other hand, the studies of Tore *et al.* and Ingenbleek *et al.* showed an association in humans between high *cysteine* intake and lower plasma homocysteine.^38,40^ An explanation for this might be the study of Singh *et al.*, which shows that the reaction of CBS plays a crucial role in the trans-sulfuration pathway of sulfur amino acids, catalysing cysteine and homocysteine into cystathionine and H_2_S.^99^ Promoting this reaction via increased cysteine intake might prevent an overload of homocysteine and its intracellular excretion into the bloodstream.^100^ These data demonstrate that *methionine* and *cysteine*, both SAAs, may have opposing effects; thus, it is important to specify and distinguish them in future studies assessing a potential therapeutic or toxic effect. Furthermore, no study has been conducted with supplementation of only cysteine in humans. All the data come from the analysis of diet intake, and thus, more clinical studies are needed to confirm the beneficial effect of cysteine.

The classification of *hydrophobic amino acids* can be divided into two different subgroups: the aromatic amino acids *phenylalanine*, *tryptophan*, and *tyrosine*^93^ and the BCAA *isoleucine*, *leucine*, and *valine*.^92^ The aromatic amino acids group promotes in CKD patients the accumulation of uraemic toxins like p-cresyl sulfate and indoxyl sulfate,^93^ while the BCAA cause myocardial injury *in vivo*, thereby increasing the risk for cardiovascular events.^92^ Nonetheless, the effect of BCAA supplementation is still ambiguous, as some *in vivo* studies report preventive^78,79^ and others adverse^91,92^ effects. Here, a potential mediator for the effect of BCAA might be the mammalian target of rapamycin (mTOR),^79,91^ which already received attention from studies outside of the systematic review.^101–103^ Further investigation of BCAA supplementation is needed to understand this complex mechanism that might require distinction between leucine, isoleucine, and valine. Although the studies have been carried out in different organisms, this differentiation could reflect their different biochemical properties due to their side chain. Further investigation of these subgroups will provide novel insights into their specific functions and contributions to the observed adverse effects (*Figure 6–7*; Supplementary material online, *Table S4*). For example, phenylalanine, tryptophan, and tyrosine are commonly described as aromatic amino acids,^93^ but they could be considered as distinct types of aromatic amino acids based on their metabolism: phenylalanine can be converted into tyrosine, which can then be metabolized to produce p-cresyl sulfate. On the other hand, tryptophan cannot be converted from the other two aromatic amino acids,^104^ and is a precursor of indoxyl sulfate,^105^ highlighting that the generalization of amino acid groups might not always be useful.

Finally, it is important to note that although *arginine* causes the highest number of preventive effects, both *in vivo*^24,47,51,53–56,58,59^ and in clinical studies,^60–62^ this amino acid has also been reported to cause multiple adverse effects when supplemented long-term.^82–84^ Therefore, the effect of arginine and how the duration of supplementation impacts the outcome require further investigation. In this regard, *citrulline* is an essential cofactor, as citrulline supplementation increases the bioavailability of arginine *in vivo*^47,51^ and it may be considered to improve the outcome of long-term arginine supplementation in patients.

Special attention has to be drawn to the doses used in the different studies: the dosage of all *in vivo* studies (see Supplementary material online, *Table S5*) was comparable to the normal daily human intake,^106–109^ only four studies used 10- to 20-times higher doses, showing preventive effects for arginine^53,54^ and taurine^74^ and adverse effects for methionine^87^ (the human equivalent dosage was calculated using the formula by Reagen-Shaw *et al.*^110^) The effects of the high doses have to be carefully re-evaluated to prevent any undetected harmful effects, before any translation to the clinical setting can be made. One study used 10-times lower cysteine intake, causing, together with sub-chronic methionine supplementation, adverse effects.^85^ The concentration used in the clinical studies supplementing amino acids (see Supplementary material online, *Table S6*) was similar to the reference values of a daily amino acid intake.^106–109^ Therefore, all studied with a calculated human equivalent dose are comparable in regard of the dose used, except from the few exceptions mentioned before, and point to amino acids as interesting mediators for human translation with dosage in physiological conditions and thus decreasing the possible side effects of the treatment.

One of the most notable findings of this systematic review is the limited availability of clinical research evaluating amino acid supplementation, with a particular shortage of high-quality RCTs. Out of the 13 clinical studies included in this systematic review, only five of them were randomized controlled trials. These RCTs focused only on the supplementation of arginine,^60,84^ lysine:arginine ratio,^62^ glutamine,^45^ and aspartate and glutamate.^70^ From these five studies four showed preventive effects from the amino acids (arginine, lysine:arginine ratio, aspartate and glutamate, as well as glutamine), while only the long-term supplementation of arginine has shown adverse effects.^84^ Overall, RCT studies showed a positive effect for AA supplementation except for the study by *Wilson et al*.^84^ They express concerns about long-term supplementation of arginine. While this concern should not be underestimated, they also showed that the plasma concentration of arginine increased only by 10 nmol/L, which is a minimal increase since the physiological concentration typically lies in the range of 50–100 µmol/L.^111–113^ Here, it would be interesting to investigate how the arginine plasma concentration changes over time, not just between baseline and endpoint. Nonetheless, the possible problematic outcome for long-term supplementation of all amino acids has to be carefully investigated before clinical implementation.

From the clinical studies, only six publications supplemented amino acids (arginine,^60,61,84^ arginine & lysine,^62^ aspartate and glutamate,^70^ and glutamine,^45^) enabling direct analysis of their effect and insights to their mode of action. The other seven publications analysed the diet composition of the study group, evaluated their (specific) amino acid intake, or looked for association and correlation with cardiovascular and renal comorbidities. While this method is quite useful for understanding whether amino acids have a positive or negative correlation with specific diseases, these studies lack the important aspect of analysing the mode of action of amino acid supplementation. Another limitation is the sex bias, from the clinical studies, five have equal number of participants from each sex, while three are only performed in males and only one in females, the rest had an uneven number between male and female participants. Moreover, in the *in vivo* studies, there is a sex bias, 21 publications result from male animals, nine from female animals, and two from both sexes, making the translation to the population even more difficult. Future studies should always consider both sexes, since there are sex differences in the progression of CVD and CKD: men are more prone to develop CVD, but the mortality for women is higher.^114^ On the other hand, women suffer more from CKD than men, even though men reach kidney failure sooner.^115^ One more limitation is the concept of ‘amino acid supplementation’ englobes orally, by injection, or via media *in vitro,* indicating that not all publications in this systematic review should have the same impact. Nonetheless, most of the publications described in this systematic review analysed *in vivo* supplementation, although with different animal models. However, the translation of most results to humans is still pending.

Unfortunately, a meta-analysis to further strengthen the data of this systematic review was not possible, due to the wide number of different amino acids, species, supplementation times and concentrations used, leading to small numbers of publications per condition. This systematic review builds the basis for future investigation in CVD–CKD by giving an overview of the impact of amino acid supplementation on processes underlying their comorbidities, resulting in preventive or adverse effects. The importance of amino acids supplementation extends beyond the potential impact on CVD and CKD, for example their effect in other tissues like liver, skeletal muscles and other diseases like diabetes and obesity might be of relevance. This influence has already been investigated by some authors. For example, arginine and glycine were shown to influence lipid metabolism in the liver of rats,^53,57^ while aspartate and glutamate were observed to inhibit the development of fatty liver disease in cholesterol-fed rabbits^69^; these effects, if further validated, might improve the outcome of atherosclerosis.^53,57,69,78,88^ Homoarginine,^64^ taurine,^73^ and SAAs^40^ have been connected to obesity: Taurine prevents abdominal fat deposition while preserving endothelial function in the thoracic aorta of hypothalamic obese rats,^73^ while SAA intake was linked to obesity and cardiometabolic disorder in humans.^40^ Homoarginine plasma concentration was shown to be reduced in obese ZSF1 rats,^64^ but a correlation to humans is still pending.^116^ On the other hand, skeletal muscle degradation, commonly observed in CKD patients and considered a new risk factor for CVD,^117^ was shown to be reduced in an adenine-CKD rat model by lysine supplementation.^63^ Another significant contributor to cardiac and renal diseases is the impact of neural hormones, an aspect that remains understudied in this systematic review, with only glutamine, aspartate and glutamate, and homoarginine showing a reduction in brain natriuretic peptide (BNP).^44,65,70^ These relations to other diseases should be addressed in future clinical studies.

This systematic review demonstrates that amino acids are essential not only for protein synthesis, but also for many different signalling pathways involved in the onset and progression of cardiovascular and renal diseases. The data presented in his review focussing on preventive effects of amino acid supplementation outnumbering potential adverse effects, although it is important to assess which pathways are particularly regulated and more clinical studies, with different CVD and CKD populations englobing both sexes are required to corroborate the beneficial effects observed in the *in vivo* studies. Nevertheless, our findings suggest a favourable risk-benefit profile, for amino acid supplementation as a therapeutic strategy.

## 5. Perspective

Altogether, the results of this systematic review point to specific amino acids as targets for preventing and treating CVD and CKD. However, most of the publications included in this systematic review were performed *in vivo* in various animal models, and the translation to humans is still pending. In this regard, only a limited number of clinical trials have been found regarding amino acid supplementation, pointing to this as a key gap and future perspective. The current challenge to accelerate the insight of amino acid supplementation in clinical trials, is to shift the focus away from high vs. low protein diets and towards studying the effect of supplementing individual amino acids. This would greatly improve the understanding of how each amino acid affect the cardiovascular and renal health. The importance of focusing on individual amino acids becomes especially evident when examining *the* SAAs, as *cysteine* and *methionine* have strongly opposite effects yet are often mentioned as a single group. This would not only result in more transparent research, but cloud also improve the outcome of future studies, preventing otherwise negative or no outcome at all. Moreover, in the future, assessing the underlying metabolic status of the single amino acids in routinely check-ups could improve disease detection and outcome, allowing for possible effective personalized medicine.

## Supplementary Material

cvag007_Supplementary_Data

## Data Availability

The data underlying this article will be shared on reasonable request to the corresponding author.
