# Supplementary material for: Impact of amino acid supplementation on cardiovascular and chronic kidney diseases: a systematic review
Source: Cardiovasc Res. 2026 Jan 21;122(2):178–94. doi: 10.1093/cvr/cvag007 (PMC13017769; doi:10.1093/cvr/cvag007)
Supplement: cvag007_Supplementary_Data [file cvag007_supplementary_data.zip › Supplement_clean version_Rev2.docx]

**Impact of amino acid supplementation on cardiovascular and chronic kidney diseases: a systematic review**

Dustin Mikolajetz, MSc.^a)^, Sahir Kalim, MD^b)^, Heidi Noels, PhD^a)^, Zhuojun Wu, PhD^a)^, Vera Jankowski, Prof^a)^, Joachim Jankowski, Prof^a)c)*)^, Sofía de la Puente-Secades, PhD^a)*)^

1. Institute of Molecular Cardiovascular Research, Uniklinik RWTH Aachen, Germany
2. Department of Medicine, Division of Nephrology, Massachusetts General Hospital and Harvard Medical School, Boston, Massachusetts
3. Experimental Vascular Pathology, Cardiovascular Research Institute Maastricht (CARIM), University of Maastricht, The Netherlands

*) Authors contributed equally

***Amino acid supplementation on CVD and CKD***

**Correspondence to:**

Prof. Dr. Joachim Jankowski

RWTH University of Aachen | Institute for Molecular Cardiovascular Research

Pauwelsstrasse 30 | 52074, Aachen | Germany

Tel.: (+49)-241-80-80580 | Fax: (+49)-241-80-52716

Email: [jjankowski@ukaachen.de](mailto:jjankowski@ukaachen.de)

**List of contents**

**Supplementary Table S1: Risk of bias assessment.** *In vitro* studies were assessed with the QUIN, *in vivo* studies with SYRCLE, and clinical studies with modified downs and black tool and categorized into low, medium and high risk.

**Supplementary Table S2: Amino acid groups and their effects on CVD and CKD**. **CKD**. Amino acids group: hydrophilic uncharged, hydrophilic cationic, hydrophilic anionic, sulphur containing, and hydrophobic with preventive or adverse effects described for CVD and CKD.

**Supplementary Table S3: Preventive effects of amino acid supplementation.** Summary of preventive effects of amino acids supplementation, mechanism, comorbidity, type of study and reference.

**Supplementary Table S4: Adverse effects of amino acid supplementation.** Summary of adverse effects of amino acids supplementation, mechanism, comorbidity, type of study and reference.

**Supplementary Table S5: Human equivalent dose calculated after equation from Reagen-Shaw *et al*.** Human equivalent dose could only be calculated when daily intake was given and calculated for human weight of 70 kg.

**Supplementary Table S6: Overview of all clinical studies included in the systematic review.** Information about type of study, amino acid, study -name, -duration, -population, amino acid dosage, total intake and main outcome of studies are mentioned.

**Supplementary Table S1: Risk of bias assessment.** *In vitro* studies were assessed with the QUIN, *in vivo* studies with SYRCLE, and clinical studies with modified downs and black tool and categorized into low, medium and high risk.

| Publication (Year) | Tool | Risk of bias |
| --- | --- | --- |
| Alam *et al*. (2013)^54^ | SYRCLE | medium risk |
| Ananthakrishnan *et al*. (2009)^48^ | SYRCLE | medium risk |
| Andrews *et al*. (2024)^88^ | SYRCLE | medium risk |
| Barba *et al*. (2021)^93^ | SYRCLE | medium risk |
| Berg *et al*. (2013)^24^ | QUIN | medium risk |
| Biswas *et al.* (2025)^96^ | SYRCLE | medium risk |
| Brawley *et al*. (2004)^76^ | SYRCLE | medium risk |
| Büttner *et al*. (2022)^64^ | SYRCLE | medium risk |
| Cojocaru *et al*. (2021)^80^ | SYRCLE | low risk |
| Costa *et al*. (2013)^59^ | SYRCLE | medium risk |
| Gemmel *et al*. (2021)^49^ | SYRCLE | medium risk |
| Grandvuillemin *et al*. (2018)^56^ | QUIN | low risk |
| Huang *et al*. (2020)^82^ | SYRCLE | medium risk |
| Ingenbleek *et al*. (2012)^38^ | Modified Downs and Black* | medium risk |
| Jackson *et al*. (2002)^77^ | SYRCLE | medium risk |
| Jiang *et al*. (2021)^92^ | SYRCLE | medium risk |
| Kaya *et al*. (2023)^58^ | SYRCLE | low risk |
| Koch *et al*. (2022)^65^ | SYRCLE | low risk |
| Kostic *et al*. (2019)^85^ | SYRCLE | low risk |
| Larsson *et al*. (2015)^43^ | Modified Downs and Black* | low risk |
| Leão *et al*. (2019)^73^ | SYRCLE | medium risk |
| Li *et al*. (2018)^87^ | SYRCLE | low risk |
| Lobel *et al*. (2020)^72^ | SYRCLE | medium risk |
| Mensegue *et al*. (2020)^74^ | SYRCLE | low risk |
| Mirmiran *et al*. (2017)^39^ | Modified Downs and Black* | low risk |
| Morita *et al*. (2014)^51^ | SYRCLE | low risk |
| Mouton *et al*. (2024)^44^ | SYRCLE | medium risk |
| Murthy *et al*. (2005)^89^ | SYRCLE | medium risk |
| Napoli *et al*. (2006)^55^ | SYRCLE | medium risk |
| Nitz *et al*. (2022)^66^ | SYRCLE | medium risk |
| Ozcan *et al*. (2022)^52^ | SYRCLE | medium risk |
| Parsanathan *et al*. (2018)^71^ | QUIN | medium risk |
| Pires *et al*. (2021)^45^ | Modified Downs and Black | low risk |
| Rom *et al*. (2022)^75^ | SYRCLE | medium risk |
| Rosenberger *et al*. (2011)^86^ | SYRCLE | medium risk |
| Rossi *et al*. (2015)^94^ | Modified Downs and Black* | medium risk |
| Schlaich *et al*. (2004)^61^ | Modified Downs and Black* | medium risk |
| Schwartz *et al*. (2007)^95^ | SYRCLE | medium risk |
| Shimomura *et al*. (2014)^63^ | SYRCLE | medium risk |
| Shivgunde *et al*. (2023)^50^ | SYRCLE | low risk |
| Siasos *et al*. (2009)^60^ | Modified Downs and Black | medium risk |
| Sim *et al*. (2016)^46^ | QUIN | medium risk |
| Souza *et al*. (2019)^83^ | SYRCLE | medium risk |
| Temizturk *et al*. (2021)^70^ | Modified Downs and Black | medium risk |
| Tore *et al*. (2023)^40^ | Modified Downs and Black* | low risk |
| Tsuboi *et al*. (2018)^47^ | SYRCLE | medium risk |
| Tuttle *et al*. (2012)^41^ | Modified Downs and Black* | medium risk |
| Vallabha *et al*. (2016)^53^ | SYRCLE | medium risk |
| Vega-López *et al*. (2010)^62^ | Modified Downs and Black | medium risk |
| Venkatesh *et al*. (2017)^57^ | SYRCLE | low risk |
| Virtanen *et al*. (2006)^42^ | Modified Downs and Black* | low risk |
| Wang *et al*. (2021)^81^ | SYRCLE | low risk |
| Wilson *et al*. (2007)^84^ | Modified Downs and Black | medium risk |
| Witham *et al*. (2013)^79^ | SYRCLE | low risk |
| Yanni *et al*. (2003)^67^ | SYRCLE | medium risk |
| Yanni *et al*. (2005)^68^ | SYRCLE | medium risk |
| Yanni *et al*. (2010)^69^ | SYRCLE | medium risk |
| Yoshida *et al*. (2018)^90^ | SYRCLE | medium risk |
| Zhang *et al*. (2020)^91^ | SYRCLE | medium risk |
| Zhao *et al*. (2016)^78^ | SYRCLE | medium risk |
| Modified Downs and Black*: clinical study without intervention (questions for intervention were disregarded) | | |

**Supplementary Table S2: Amino acid groups and their effects on CVD and CKD**. Amino acids group: hydrophilic uncharged, hydrophilic cationic, hydrophilic anionic, sulphur containing, and hydrophobic with preventive or adverse effects described for CVD and CKD.

| **Amino acid group** | **Preventive** | | **Adverse** | |
| --- | --- | --- | --- | --- |
|  | **CVD** | **CKD** | **CVD** | **CKD** |
| Hydrophilic uncharged | Yes | Yes | No | No |
| Hydrophilic cationic | Yes | Yes | Yes | Yes |
| Hydrophilic anionic | Yes | No | Yes | No |
| Sulphur containing | Yes | Yes | Yes | Yes |
| Hydrophobic | Yes | Yes | Yes | Yes |

CKD: chronic kidney disease, CVD: cardiovascular disease

**Supplementary Table S3: Preventive effects of amino acid supplementation**. Summary of preventive effects of amino acids supplementation, mechanism, comorbidity, type of study and reference.

| **Amino acids supplemented** | **Mechanism** | **Comorbidity** | **Type of study** | | | **Ref.** |
| --- | --- | --- | --- | --- | --- | --- |
|  |  |  | ***in vitro*** | ***in vivo***  **(animals)** | **clinical** |  |
| Glutamine | Prevents carbamylation | Uremic toxin | X |  |  | ^24^ |
| Glutamine | Increases HDL-C, PON-1, PRx, GPx; decreases ROS | Atherosclerosis |  |  | X | ^45^ |
| Glutamine | Increases Myh6, decreases Myh7 in MI, improving LV remodelling | Myocardial infarction |  | X |  | ^44^ |
| Serine | Reduces homocysteine uptake | Atherosclerosis | X |  |  | ^46^ |
| Threonine | Reduces SBP | Blood pressure |  |  | X | ^41^ |
| Citrulline | Reduces Arginase 2 activity, increases eNOS and NO, decreases endothelial senescence | Atherosclerosis |  | X |  | ^47^ |
| Citrulline | Converted by AS+AL into arginine, increases NO | Blood pressure |  | X |  | ^48^ |
| Citrulline | Increases glycocalyx volume and improves NO-dependant relaxation | Blood pressure |  | X |  | ^49^ |
| Citrulline | Decreases ROS | Blood pressure |  | X |  | ^50^ |
| Citrulline | Decreases XOX activity, decreases ROS | Blood pressure |  | X |  | ^52^ |
| Arginine | Increases NO; reduces TG levels and ACE activity, reduces SBP | Blood pressure |  | X |  | ^53^ |
| Arginine | Increases NO, reduces ROS, prevents obesity-related changes in heart, liver, and pancreas | Metabolic disorder |  | X |  | ^54^ |
| Arginine (+ antioxidant) | Increases NO, reduces ROS, reduces atherosclerotic plaque area | Atherosclerosis |  | X |  | ^55^ |
| Arginine (+low protein diet) | Restores vasodilation in aortic rings, presumably via increased NO | Blood pressure | X |  |  | ^56^ |
| Arginine + citrulline (under high glucose) | Increases eNOS activity, increases NO, restores endothelial function and delays cellular senescence | Atherosclerosis |  | X |  | ^47^ |
| Arginine + citrulline | Increase in plasma arginine, increases NO_x_ and cGMP | Atherosclerosis |  | X |  | ^51^ |
| Arginine | Decreases sICAM, reduces PWV | Atherosclerosis |  |  | X | ^60^ |
| Arginine (+hypercholesterolemic diet) | Reduces cholesterol | CVD events |  | X |  | ^57^ |
| Arginine | Reduces ROS, apoptosis, inflammation, and mitochondrial dynamics, increases Otulin | Myocardial infarction |  | X |  | ^58^ |
| Arginine | Increases response to ACh and forearm blood flow | Blood pressure |  |  | X | ^61^ |
| Arginine | Decreases VLDL-C and TG | CVD events |  |  | X | ^62^ |
| Arginine (+ radiation) | Preserves VEGF and FGF | CVD events |  | X |  | ^59^ |
| Arginine | Prevents carbamylation | Uremic toxin | X |  |  | ^24^ |
| Lysine | Decreases iPTH, increases alanine and proline (reduce apoptosis), increases arginine and homoarginine (reduce mineral precipitation) | Calcification |  | X |  | ^63^ |
| Lysine | Prevents carbamylation | Uremic toxin | X |  |  | ^24^ |
| Histidine | Decreased blood pressure | Blood pressure |  |  | X | ^41^ |
| Histidine | Prevents carbamylation | Uremic toxin | X |  |  | ^24^ |
| Homoarginine | Improves right ventricular function in HFpEF | CVD events |  | X |  | ^64^ |
| Homoarginine (in 5/6 Nx) | Reduce cardiac remodelling, inhibits TNAP, improves EF | CVD events |  | X |  | ^65^ |
| Homoarginine | Inhibits Myh9 in T-cells, decreases T-cell mobility, activation and proliferation | Atherosclerosis |  | X |  | ^66^ |
| Aspartate + glutamate (in del Nido solution) | Reduce leukocyte accumulation, troponin-I and pro-BNP, increases left ventricular function | Myocardial infarction |  |  | X | ^70^ |
| Aspartate + glutamate | Reduce formation of fatty streaks and foam cell formation | Atherosclerosis |  | X |  | ^67^ |
| Aspartate + glutamate | Increases HDL-C, preserves ApoA1 | Atherosclerosis |  | X |  | ^68^ |
| Aspartate + glutamate | Reduces γ-GT, prevents ox-LDL | Atherosclerosis |  | X |  | ^69^ |
| Sulphur amino acids (+ high protein diet) | Reduces homocysteine | CVD events |  |  | X | ^38^ |
| Cysteine | Increases G6PD, GSH, reduces NOX and ROS | Atherosclerosis | X |  |  | ^71^ |
| Cysteine | Reduces homocysteine | CVD events |  |  | X | ^40^ |
| Cysteine | Increases GSH | Metabolic disorder |  |  | X | ^40^ |
| Cysteine | Reduces stroke | CVD events |  |  | X | ^43^ |
| Cysteine and methionine | Modify gut tryptophanase, reducing production of indole | Uremic toxin |  | X |  | ^72^ |
| Cysteine | Prevents carbamylation | Uremic toxin | X |  |  | ^24^ |
| Taurine | Reduces perigonadal fat deposition, TG and MDA | Metabolic disorder |  | X |  | ^73^ |
| Taurine (during pregnancy) | Reduces SBP, increases eNOS, Bax/Bcl2, preventing aortic remodelling (in adult offspring) | Hypertension |  | X |  | ^74^ |
| Taurine | Prevents carbamylation | Uremic toxin | X |  |  | ^24^ |
| Glycine | Reduces homocysteine | Atherosclerosis | X |  |  | ^46^ |
| Glycine | Decreases ROS, reduces plaque area | Atherosclerosis |  | X |  | ^75^ |
| Glycine (+hypercholesterolemic diet) | Reduces cholesterol, reduces plasma homocysteine, increases nitrite/nitrate level | Atherosclerosis |  | X |  | ^57^ |
| Glycine (during pregnancy + diet restriction) | Reversed impaired arteries relaxation, increases NO release | Blood pressure |  | X |  | ^76^ |
| Glycine (+low protein diet) | Reduces blood pressure in offspring | Blood pressure |  | X |  | ^77^ |
| Glycine | Prevents carbamylation | Uremic toxin | X |  |  | ^24^ |
| Leucine | Increases Abcg5, Abcg8, decreases LDL-C, increases HDL-C, decreasing inflammation, decreases atherosclerotic lesion area | Atherosclerosis |  | X |  | ^78^ |
| Leucine | Activates mTOR, ERK1/2, reduces cardiac fibrosis and inflammation, reduces cardiac remodelling | Myocardial infarction |  | X |  | ^79^ |
| Leucine (+high cholesterol) | Decreases TG | Atherosclerosis |  | X |  | ^80^ |
| Leucine | Prevents carbamylation | Uremic toxin | X |  |  | ^24^ |
| Valine (+high cholesterol) | Decreases TG | Atherosclerosis |  | X |  | ^80^ |
| Valine | Prevents carbamylation | Uremic toxin | X |  |  | ^24^ |
| Phenylalanine (+high salt diet) | Increases BH4, NO and reduces superoxide and blood pressure | Blood pressure |  | X |  | ^81^ |
| Alanine | Prevents carbamylation | Uremic toxin | X |  |  | ^24^ |
| Proline | Prevents carbamylation | Uremic toxin | X |  |  | ^24^ |
| Tryptophan | Prevents carbamylation | Uremic toxin | X |  |  | ^24^ |

*Abcg5:* ATP-binding cassette sub-family G member 5*, Abcg8:* ATP-binding cassette sub-family G member 8**,** ACE: angiotensin 1 converting enzyme, ACh: acetylcholine, AL: argininosuccinate lyase, ApoA1: apolipoprotein A1, AS: argininosuccinate synthetase, BH4: tetrahydrobiopterin, cGMP: cyclic guanosine monophosphate, CVD: cardiovascular disease, EF: ejection fraction, eNOS: endothelial nitric oxide synthase, ERK: extracellular signal-regulated kinase, FGF: fibroblast growth factor, G6PD: glucose-6-phosphate dehydrogenase, GPx: glutathione peroxidase, GSH: glutathione, HDL-C: high-density lipoprotein-cholesterol, HFpEF: heart failure with preserved ejection fraction, iPTH: intact parathyroid hormone, LV: left ventricular, MDA: malondialdehyde, MI: myocardial infarction, mTOR: mammalian target of rapamycin, Myh: myosin heavy chain, NO: nitric oxide, NO_x_: nitric oxides, NOX: NADPH oxidase, ox-LDL: oxidised low-density lipoproteins, PON‑1: paraoxonase, Pro-BNP: pro-brain natriuretic peptide, PRx: total peroxidase, PWV: pulse wave velocity, ROS: reactive oxygen species, SBP: systolic blood pressure, sICAM: soluble intercellular adhesion molecule, TG: triglyceride, TNAP: tissue-nonspecific alkaline phosphatase, VEGF: vascular endothelial growth factor, VLDL-C: very low-density lipoprotein cholesterol, XOX: xanthine oxidase, γ-GT: gamma glutamyl transferase.

**Supplementary Table S4: Adverse effects of amino acid supplementation.** Summary of adverse effects of amino acids supplementation, mechanism, comorbidity, type of study and reference.

| **Amino acids supplemented** | **Mechanism** | **Comorbidity** | **Type of study** | | | **Ref.** |
| --- | --- | --- | --- | --- | --- | --- |
|  |  |  | ***in vitro*** | ***in vivo*** | **clinical** |  |
| Arginine (long-term) | Reduces or fails to improve NO, does not improve walking distance in PAD | Atherosclerosis |  |  | X | ^84^ |
| Arginine (long-term) | Increases urinary albumin-creatinine ratio | Renal ageing |  | X |  | ^82^ |
| Arginine (+resistance training) | Increased urea levels | Uremic toxins |  | X |  | ^83^ |
| Glutamate | Increased association with CVD | CVD events |  |  | X | ^39^ |
| Glutamate | Increased association with systolic blood pressure | Blood pressure |  |  | X | ^41^ |
| Cysteine (+subchronic methionine) | Decreases vitamin B12, fibrinogen, vWF concentration and activity, increases D-dimer, dilation of microcirculation, intercellular edema | CVD events |  | X |  | ^85^ |
| Homocysteine | Increases ER stress, endothelial cell death | Atherosclerosis | X |  |  | ^46^ |
| Homocysteine | Increases MMP2 and 9, decreases connexin 43 and 45, reduces cardiac conduction | CVD events |  | X |  | ^86^ |
| Methionine (subchronic) | Decreases vitamin B12, vWF activity, dilation of microcirculation | CVD events |  | X |  | ^85^ |
| Methionine | Causes hyperhomocysteinemia& increases SBP | Blood pressure |  | X |  | ^87^ |
| Methionine (+reduced methyl donors & vitamins) | Decreases SAM/SAH ratio, increases atherosclerotic plaque burden | Atherosclerosis |  | X |  | ^88^ |
| Methionine | Increases hyperplasia after vessel injury | Atherosclerosis |  | X |  | ^89^ |
| Methionine (under diet restriction) | Inhibits trans-sulphuration pathway, increase oxidative stress, lower Klotho expression | Renal ageing |  | X |  | ^90^ |
| Methionine | Increases total cysteine (trend), associated with high BMI and whole-body fat | Metabolic disorder |  |  | X | ^40^ |
| Methionine | Decreases glutathione | Metabolic disorder |  |  | X | ^40^ |
| Methionine | Increased association with SBP and DBP | Blood pressure |  |  | X | ^41^ |
| Methionine | Increases association with CVD | CVD events |  |  | X | ^42^ |
| Alanine | Increased association with SBP and DBP | Blood pressure |  |  | X | ^41^ |
| Phenylalanine | Increased association with DBP | Blood pressure |  |  | X | ^41^ |
| Proline | Increased association with CVD | CVD |  |  | X | ^39^ |
| BCAA (isoleucine + leucine + valine) | Increases ROS, AMPK-ULK-1 pathway, autophagy in myocardial tissue | CVD |  | X |  | ^92^ |
| Leucine (+cholesterol) | Activated mTORC1, inhibiting mitophagy, promoting apoptosis | Atherosclerosis |  | X |  | ^91^ |
| Aromatic AA (phenylalanine + tryptophan + tyrosine) | Increases p-CS and IS | Uremic toxins |  | X |  | ^93^ |
| High protein intake (focus on aromatic AA) | Increased protein/fibre ratio | Uremic toxins |  |  | X | ^94^ |
| Arginine* | No effect on cholesterol levels |  |  | X |  | ^95^ |
| Glycine* | Decreases HDL-C and TG |  |  | X |  | ^96^ |

*The study described neither harmful nor protective effects.

AMPK: AMP-activated protein kinase, BMI: body mass index, CVD: cardiovascular disease, DBP: diastolic blood pressure, ER: endoplasmic reticulum, HDL-C: high-density lipoprotein-cholesterol, IS: indoxyl sulphate, MMP: matrix metalloproteinase, mTORC1: mammalian target of rapamycin complex 1, NO: nitric oxide, PAD: peripheral artery disease, p-CS: para-cresyl sulphate, ROS: reactive oxygen species, SAH: S-adenosylhomocysteine, SAM: S-adenosylmethionine, SBP: systolic blood pressure, TG: triglyceride, ULK1: UNC-51 like kinase 1, vWF: von Willebrand factor.
